# Analysis of correlation-based biomolecular networks from different omics data by fitting stochastic block models

**DOI:** 10.12688/f1000research.18705.2

**Published:** 2019-08-27

**Authors:** Katharina Baum, Jagath C. Rajapakse, Francisco Azuaje

**Affiliations:** 1 Bioinformatics and Modelling, Luxembourg Institute of Health, Strassen, Luxembourg; 2 Mathematical Modelling of Cellular Processes, Max Delbrück Center for Molecular Medicine in the Helmholtz Association, Berlin, Germany; 3 School of Computer Science and Engineering, Nanyang Technological University, Singapore, Singapore

**Keywords:** biomolecular networks, co-expression networks, edge relevance, hierarchical stochastic block model, missing and spurious edges, module detection, network-based omics analysis

## Abstract

**Background:** Biological entities such as genes, promoters, mRNA, metabolites or proteins do not act alone, but in concert in their network context. Modules, i.e., groups of nodes with similar topological properties in these networks characterize important biological functions of the underlying biomolecular system. Edges in such molecular networks represent regulatory and physical interactions, and comparing them between conditions provides valuable information on differential molecular mechanisms. However, biological data is inherently noisy and network reduction techniques can propagate errors particularly to the level of edges. We aim to improve the analysis of networks of biological molecules by deriving modules together with edge relevance estimations that are based on global network characteristics.

**Methods: **The key challenge we address here is investigating the capability of stochastic block models (SBMs) for representing and analyzing different types of biomolecular networks. Fitting them to SBMs both delivers modules of the networks and enables the derivation of edge confidence scores, and it has not yet been investigated for analyzing biomolecular networks. We apply SBM-based analysis independently to three correlation-based networks of breast cancer data originating from high-throughput measurements of different molecular layers: either transcriptomics, proteomics, or metabolomics. The networks were reduced by thresholding for correlation significance or by requirements on scale-freeness.

**Results and discussion:** We find that the networks are best represented by the hierarchical version of the SBM, and many of the predicted blocks have a biologically and phenotypically relevant functional annotation. The edge confidence scores are overall in concordance with the biological evidence given by the measurements. We conclude that biomolecular networks can be appropriately represented and analyzed by fitting SBMs. As the SBM-derived edge confidence scores are based on global network connectivity characteristics and potential hierarchies within the biomolecular networks are considered, they could be used as additional, integrated features in network-based data comparisons.

## Introduction

High-throughput measurement techniques are advancing and become ever less expensive, enabling the screening of multiple biological data layers in single patients as almost standard clinical diagnostic tools. The wealth of the biological data can only be understood if treating the measured entities – gene promotors, mRNA, metabolites, proteins and their activity – not separately, but in their network context ^1^. Thereby, one method to capture the interdependencies of the intracellular machinery relies on the hypothesis that strongly connected molecular entities are either co-activated or co-repressed, i.e. their measured abundances should be correlated ^2– 4^. Fully connected, weighted biomolecular networks can be established, in which each node corresponds to a molecular entity and is connected to each other node by an edge. The weight of the edge is the correlation between the measurements and is considered to represent how strongly the nodes are connected, interact or regulate each other.

Approaching a network-level analysis of a biological system by correlation-based interactions has the advantage that it does not require *a priori* knowledge, and thus it is focused on the interaction profile for which evidence can be found in the measurements of the considered condition. However, this is a blessing and a curse: correlation-based networks suffer from the fact that the biological measurements are inherently noisy, even more so for small sample sizes as in rare diseases or in personalized medicine. This affects the values of the edge weights and the results of network reduction, and can be deleterious for subsequent analyses of the networks. In these cases, considering in addition alternative sources of edge relevance that are based on more global characteristics of the system might be beneficial and could make the network representations of the system and their analysis more robust.

One of the commonly performed analyses of correlation-based networks relies on the observation that biological networks exhibit a modular structure, in which tightly regulated modules are loosely connected to other modules. An important readout from correlation-based networks therefore is the biological and functional characterization of condition-specific modules, i.e. communities of co-regulated entities. A plethora of methods for module detection in networks has been proposed ^5– 14^. Also for the inference of edge relevance, or edge prediction, as being tightly linked to the problem of the detection of missing or spurious edges, numerous methods have been suggested ^15– 23^. In this work, we showcase a method that is able to derive, within a single framework, modules as well as scores of edge relevance: representing the biomolecular correlation-based networks as stochastic block models (SBMs) ^24^.

SBMs are the simplest form of generative network models for community structures and can also accommodate hierarchies ^25^, which are key to convey robustness to biological function. Other generative model approaches relying on scale-freeness of the network architecture have been used for edge prediction in protein-protein interaction networks ^15^, and the SBM has been used for representing other types of networks ^17, 18, 26, 27^, but not yet for biomolecular networks. In generative network model approaches, the network is described by a stringent mathematical framework based on statistical assumptions on network characteristics. For the case of the stochastic block model, this step delivers already the modular structure of the network as the nodes are assigned to blocks according to their connectivity properties to all other nodes. In contrast to other module detection methods, blocks in the SBM are not necessarily formed by tightly intra-connected entities but by entities which interact similarly with the nodes from all other blocks. Therefore, comparing SBM-derived modules between networks representing different (e.g. biomedical) conditions could be especially informative to shed light on regulatory changes. In a second step, the mathematical representation of the networks by SBMs is exploited to estimate edge probabilities. Specifically, it is assessed whether the existence of an edge in the network improves or reduces the fit of the network to the SBM. The resulting edge confidence scores are based on global network structure and can be used as alternative measure of edge relevance.

**Figure 1.**
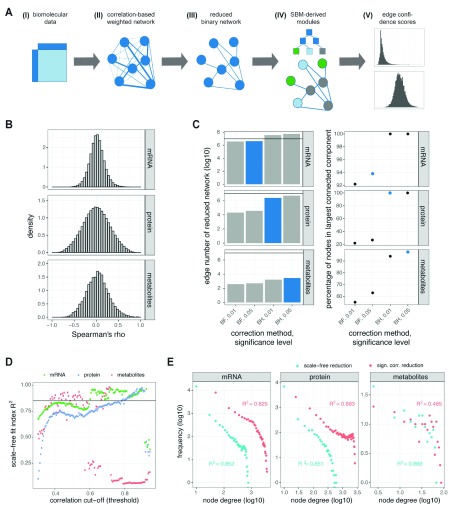
Analysis of correlation-based networks from different omics data: Pipeline and network preparation. ( **A**) Pipeline for the approach. Given the matrix of measurements of mRNA expression, protein expression or metabolite abundance for a group of samples (I), we compute a fully-connected weighted network (II) of the molecular species for each data layer separately using a correlation-based approach. We reduce the networks by setting a threshold to the edge weights and binarize the networks (III). Each network is fitted to different types of stochastic block models in which the network nodes are partitioned into blocks (IV), the best fitting model is employed for deriving SBM-based edge confidence scores (V). ( **B**) We established correlation-based networks using Spearman’s correlation within mRNA expression, protein expression and metabolite biomolecular data from a subgroup of cancer patients. Shown are histograms of the correlations obtained for all edges of the networks. ( **C**) We only kept edges in the network that had a correlation which differed significantly from zero (sign. corr.). For different multiple testing correction methods (BH: Benjamini-Hochberg, BF: Bonferroni) and significance levels (0.05, 0.01), different degrees of reduction can be achieved. We chose for each data layer the most stringent threshold (highlighted in blue) that reduced the edge count to less than 10 ^7^ edges (left) while keeping the percentage of nodes in the largest connected component high (right). ( **D**) Scale-free fit indices *R* ^2^ according to 3 for the mRNA (green), protein (blue), and metabolite (red) network reduced by different correlation thresholds between 0.3 and 0.95. The employed threshold of 0.85 for the scale-free fit index is indicated by a black line. ( **E**) Histogram of the node degrees of the networks reduced by criterion on significance of correlation (red) or on scale-freeness (blue) on double log-scale together with the scale-free fit indices *R* ^2^. Networks are the more scale-free the more linear the relationship between log-frequency and log-node-degree is.

The key challenge we address here was to investigate the capability of SBMs for representing and analyzing different types of biomolecular networks. We aimed to assess to which extent the SBM is applicable to derive useful information in terms of (i) relevant clustering as well as (ii) network-based, alternative edge scores. Therefore, we showcased the SBM-based analysis (overview in Figure 1A) for three biomolecular networks of different molecular types, derived from either transcriptomic, proteomic or metabolomics data of breast cancer tumours. We assessed which of the different versions of SBM fits each data layer best. Then, we investigated whether the SBM representation is able to capture functionally relevant structures in our biomolecular networks. In detail, we determined the agreement between the predicted blocks and independent biomolecular functional annotations from databases, compared the SBM-predicted function to breast cancer signatures and showed how to derive additional predictions from SBM-based clusters. Finally, we took advantage of the description of the networks as SBMs for the computation of an edge confidence score for each edge as measure of edge relevance. The edge confidence scores can be exploited to re-establish erroneously removed edges or to remove spurious edges, or they could serve by themselves for deriving disease-relevant differences when comparing groups of patients.

Here we showcase the SBM-based analysis (overview in Figure 1A) for networks of three different molecular types, derived from transcriptomic, proteomic and metabolomics data of breast cancer tumours. We assessed which of the different versions of SBM fits each data layer best. Then, we investigated whether the SBM representation is able to capture functionally relevant structures in our biomolecular networks. In detail, we determined the agreement between the predicted blocks and independent biomolecular functional annotations from databases. Finally, we took advantage of the description of the networks as SBMs for the computation of an edge confidence score for each edge as measure of edge relevance. The edge confidence scores can be exploited to re-establish erroneously removed edges or to remove spurious edges, or they could serve by themselves for deriving disease-relevant differences when comparing groups of patients.

All code is freely available on https://gitlab.com/biomodlih/sbm-for-correlation-based-networks.

## Methods

### mRNA, protein, metabolite data for ER- breast cancer tumors

Breast cancer mRNA expression from RNAseq was obtained from the TCGA BRCA cohort via RTCGA ^28^ downloading TCGA level 3 preprocessed BRCA files (search term: *mRNAseq_Preprocess*) on Nov 2, 2017. We used the normalized RSEM values. Protein data was obtained via the CPTAC homepage from the data generated in 29. We used the first replicate of samples measured in duplicates. We employed the unshared log ratio value for each sample to maximize reliability of protein identification. Clinical data for both TCGA and CPTAC data was retrieved and evaluated using the RTCGA package. Specifically, we used the following files for mRNA, protein and clinical data, respectively:

- *gdac.broadinstitute.org_BRCA.mRNAseq_Preprocess.Level_3.2016012800.0.0/ BRCA.uncv2.mRNAseq_RSEM_normalized_log2.txt*
- *TCGA_Breast_BI_Proteome_CDAP_Protein_Report.r3/Protein_data/CDAP/TCGA_Breast_BI_Proteome.itraq.tsv*
- *gdac.broadinstitute.org_BRCA.Merge_Clinical.Level_1.2016012800.0.0/BRCA.clin.merged.txt*.

For both mRNA and protein data, we only used samples from patients whose entry "patient.breast_carcinoma_estrogen_receptor_status" in their clinical data was "negative". In addition, we restricted our analysis to solid tumour samples, i.e. TCGA sample identifiers ending with 01. This gave rise to 237 samples with 18321 measured genes for the mRNA data and 36 samples with 10625 measured proteins for the protein data.

Metabolite data was used from the Excel file provided in 30 using the measurements of 162 metabolites in the 67 samples containing ERn in their label.

### Missing values

We removed missing values in the mRNA data by replacing them by -10 to account for the fact that they arose from logarithmizing read counts of zero. In the protein data, we removed the 2195 proteins which had more than 20% of missing values over the considered samples (i.e. more than 7 NAs among the 36 samples), resulting in 8430 measured proteins that we analyzed further. The metabolite data did not contain missing values as imputation had been performed in the original publication ^30^.

### Network generation

We used the correlation computation from the Hmisc package ^31^ (function *rcorr*) to determine Spearman’s correlation of the measurements of each pair of entities (mRNA, protein or metabolite) over all samples. Only pairwise complete observations were employed. Unless stated otherwise, we neglected self-edges.

### Network reduction

The Hmisc package ^31^ was used to determine the p-value associated to the correlation (significance of the correlation being different from zero). For each data layer, we assessed four different combinations of multiple testing correction and significance thresholds: Bonferroni or Benjamini-Hochberg ^32^ multiple testing correction combined with significance thresholds of 0.01 or 0.05. The resulting reduced networks were characterized in terms of edge count and largest connected component. For each data layer, the network was chosen that yielded a sufficient degree of reduction (< 10 ^7^ edges) to enable a sufficiently short computation time and memory consumption for the fit to SBM while maintaining at the same time a high percentage of nodes being connected to each other as would be expected in biological networks. Finally, Bonferroni correction was chosen for the mRNA network, Benjamini-Hochberg correction for the smaller protein and metabolite networks. Correlations were considered significantly different from zero for corrected p-values lower than 0.05 (mRNA, metabolite) or 0.01 (protein).

For the reduction by imposing a scale-free architecture of the reduced network, we employed the *pickHardThreshold* function of the WGCNA package ^3^ with the default requirement (0.85) on goodness of fit to a power-law degree distribution of the nodes. Given the symmetric absolute correlation matrix of the network edges, this function reduces the network by one of a given set of edge thresholds at a time and determines the scale-free fit index *R* ^2^ which lies between 0 (bad fit) and 1 (perfect fit) by comparing the resulting degree distribution of the reduced network to a power-law degree distribution. The lowest of the tested edge thresholds that gives a scale-free fit index > 0.85 is reported as estimated threshold. For the edge thresholds, we started with a grid with stepsize 0.05 between 0.3 and 0.95, refining according to the resulting estimates to vectors with stepsize 0.001 between 0.5 and 0.625 for the mRNA network, between 0.7 and 0.82 for the protein network, and between 0.3 and 0.4 for the metabolite network. Finally estimated edge correlation thresholds were 0.603 (mRNA), 0.788 (protein), and 0.375 (metabolite).

### Fit to SBM

We employed four versions of the stochastic block model (SBM) derived from three SBM types (classical, degree-corrected, hierarchical SBM) and their Bayesian description ^33, 34^. In the classical SBM ^24^, the model is fully defined by a partition *b* of the nodes into blocks and the matrix *e* = { *e _rs_*} of the numbers of edges between blocks (with *e _rr_* being double the edge count within a block for convenience). We employ microcanonical formulations which imposes hard constraints on the values of the model parameters according to the observed graph *G* ^34^. Given a graph *G* and a partition *b*, the probability that the graph was generated with the observed edge count matrix *e*, *P*( *G|b*), can be described as

P(G|b)=P(G|e,b)⋅P(e).

Thereby, *P*( *A|C*, *D*) denotes the probability of *A* given *C* and *D*. With *A* = { *A _ij_*} being the adjacency matrix of the (multi)graph *G*, with $e_{r}=\sum_{s}e_{rs}$ the number of edges adjacent to block *r*, and *n _r_* the number of nodes in block *r* according to the partition *b*, we employ

$$P(G|e,b)=\frac{\prod_{r<s}e_{rs}\prod_{r}e_{rr}\;!!}{\prod_{r}n_{r}^{e_{r}}\prod_{i<j}A_{ij}\;!\;\prod_{i}A_{ii}\;!!}$$

using the definition (2 *m*)!! = 2 *^m^ · m*!. In addition, for *n _e_* = 2 *E/*( *B*( *B*+1)) the expected total number of edges with *E* the number of edges in graph *G*, and *B* the number of non-empty blocks in partition *b* the prior distribution on the edges is given by:

$$P(e)=\frac{n_{e}^{E}}{{\left(n_{e}+1\right)}^{E+B(B+1)/2}}\cdot$$

Please refer to Peixoto, 2017 ^34^ for a detailed derivation and explanation. Note that for our networks, as we only use simple graphs and do not consider self-edges, i.e. *A _ij_* ∈ {0, 1} and *A _ii_* = 0, the product $\prod_{i<j}A_{ij}\;!\;\prod_{i}A_{ii}\;!!$ simplifies to 1. The prior for the partition *b* is derived from sampling the number of non-empty blocks *B* as *P*( *B*) = 1 */N* with *N* the number of nodes in graph *G*, then sample the distribution of block sizes *n _r_* conditioned on *B* and finally the partition *b* conditioned on the former two which yields in total ^34^:

$$P(b)=\frac{\prod_{r}n_{r}\;!}{N\;!}{\left(\begin{array}{c} N-1\\ B-1 \end{array}\right)}^{-1}\frac{1}{N}\cdot$$

A drawback of the classical SBM is that all nodes within a block are forced to have similar degrees which is not appropriate for most encountered real networks. Karrer and Newman proposed the degree-corrected SBM ^35^ that is, in addition to the partition *b* and the edge count matrix *e*, defined by a degree sequence *k* = { *k _i_*} setting a degree for each node. Using the microcanonical formulation ^34^, we have

P(G|b)=P(G|k,e,b)·P(k|e,b)·P(e).

with *P*( *e*) as above. *P*( *G|k*, *e*, *b*) is the probability of generating a graph *G* where the edge counts as well as the degree sequence is fixed to a specific value given a certain partition *b* and is given by

$$P(G|k,e,b)=\frac{\prod_{r<s}e_{rs}\;!\;\prod_{r}e_{r}r\;!!\;\prod_{i}k_{i}\;!}{\prod_{r}e_{r}\;!!}\cdot$$

The prior we employ for the degree sequence is conditioned on the degree frequencies which are in turn sampled from a uniform hyperprior ^34^:

$$P(k|e,b)=\prod_{r}\;\frac{\prod_{k}\eta_{k}^{r}\;!}{n_{r}\;!}\;\prod_{r}q{\left(e_{r},\;n_{r}\right)}^{-1}$$

with $\eta_{k}^{r}$ the number of nodes of degree *k* in block *r* and *q*( *n*, *m*) the number of partitions of *n* elements into at most *m* groups.

The Bayesian approach as detailed by Peixoto ^33, 34^ prevents overfitting but is prone to underfitting, meaning that statistically relevant structures may not be detected. In particular, the number of groups which can be resolved is limited such that small groups in very large networks can hardly be detected ^34^. A solution has been proposed via the hierarchical (or nested) SBM ^25^. Therein, the edge count matrix *e* = { *e _rs_*} is described with another SBM, i.e. the blocks of the first SBM are considered as nodes a second-level SBM which are partitioned into a second level of blocks, with the according second-level edge count matrix. The second-level SBM can again be described as another third-level SBM and so on, *L* number of times, forming a nested hierarchy of SBMs.

The joint distribution for the hierarchical microcanonical degree-corrected SBM is given by ^34^

$$P(A,k,\{e_{l}\},\{b_{l}\})=P(A|k,e_{1},b_{1})\cdot P(k|e_{1},b_{1})\cdot P(\{e_{l}\})\cdot P(\{b_{l}\})$$

for *l* ∈ {0, . . . , *L*}, with *e _l_* the edge count matrix at level *l*, *b _l_* the partition at level *l*. The according prior distributions are given as described above and in addition for the edge count by ^34^

$$P(\{e_{l}\}|\{b_{l}\})=\prod_{l=1}^{L}P(e_{l}|e_{l+1},\;b_{l})$$

and imposing the boundary conditions *B _L_* = 1 for the number of nonempty blocks at the highest level *L*, and *P*( *b _L_* ) = 1. Thereby,

$$P(e_{l}|e_{l+1},b_{l})=\prod_{r<s}{\left(\begin{array}{c} n_{r}^{l}n_{s}^{l}\\ e_{rs}^{l+1} \end{array}\right)}^{-1}\prod_{r}{\left(\begin{array}{c} n_{r}^{l}(n_{r}^{l}+1)/2\\ e_{rr}^{l+1}/2 \end{array}\right)}^{-1}$$

with $\left(\begin{array}{c} n\\ m \end{array}\right)$ the number of combinations of *m* elements with repetitions from a set of *n* elements. For the prior distribution of the hierarchical partitions, we employ

$$P(\{b_{l}\})=\prod_{l=1}^{L}P(b_{l})$$

using the equation for *P*( *b*) from above with the block and node counts for the respective level *l* and the boundary condition *B*_0_ = *N*. The hierarchical model without degree correction is obtained by replacing *P*( *A|k*, *e*_1_, *b*_1_) *· P*( *k|e*_1_, *b*_1_) by *P*( *A|e*_1_, *b*_1_).

Here, we examine four SBM versions: the classical SBM, the degree-corrected SBM, the hierarchical SBM and the degree-corrected & hierarchical SBM. For fitting the SBM to one of these four SBMs (i.e. in order to determine the most probable (hierarchical) partition given the data), we converted the adjacency matrices of the reduced networks to edge lists in *csv* format, added the disconnected nodes and fed the resulting networks as graphs into the Python graph-tool ^36^ framework. Initializing a partition according to the prior distribution of the partitions from above and using an agglomerative multi-level Markov Chain Monte Carlo algorithm ^37^, that module allows for determining a (potentially hierarchical) partition, *b*, of the network, *G*, which minimizes the description length, *DL*, of the SBM ^33^:

$$\begin{array}{l} DL=-\log_{2}P(G|\lambda ,b)-\log_{2}P(\lambda ,b)\\ \;=-\log_{2}(P(G|\lambda ,b)\cdot P(\lambda ,b))\\ \;=-\log_{2}(P(G|b)\cdot P(b))=-\log_{2}(P(b|G)\cdot P(G))\text{.} \end{array}$$

Thereby, *λ* captures all parameters of the model apart from the partition, such as the number of edges between blocks and degree distribution parameters for the degree-corrected SBM, in their planar or hierarchical version. Note that the above relationship holds only under a microcanonical formulation of the priors, i.e. hard constraints imposed on the values of the model parameters *λ* by the structure of the network *G* the SBM is fitted to (see 33, 34), which enables considering only one value for the parameter *λ* for a given partition *b* and thus

P(G|b)=P(G|λ,b)⋅P(λ).

Because the probability of the network itself, *P*( *G*), is constant for a fixed observed network, the description length is monotonously inversely related to the probability that partition *b* is responsible for the observed network *G*, *P*( *b|G*). Therefore, finding the partition which minimizes the description length is equivalent to finding the partition with maximal posterior probability, *P*( *b|G*). Furthermore, as can be seen in its second term in the first line, the description length *DL* contains a "penalty" term for the complexity of the SBM description. Thus, it can be used to distinguish which of the four examined SBM types (classical, hierarchical, degree-corrected, hierarchical+degree-corrected) is most suitable to describe the network *G*. Due to the large sizes of the networks, sampling over the posterior distribution is costly. Therefore, we decided to compare different SBM versions using only the estimated maximum of the posterior, i.e. at the partition *b* with the lowest minimal description length, in the expression of the posterior odds ratio ^33^. Assuming that both SBM types that are compared, *SBM*_1_ and *SBM*_2_, are equally probable *a priori*, *P*( *SBM*_1_) = *P*( *SBM*_2_), we obtain for the posterior odds ratio Λ ^33^:

$$\begin{array}{l} \wedge =\frac{P(b_{1},\;SBM_{1}|G)}{P(b_{2},\;SBM_{2}|G)}=\frac{P(G|b_{1},\;SBM_{1})P(b_{1})P(SBM_{1})}{P(G|b_{2},\;SBM_{2})P(b_{2})P(SBM_{2})}\\ \;=\frac{P(G|b_{1},\;SBM_{1})P(b_{1})}{P(G|b_{2},\;SBM_{2})P(b_{2})}=2^{-(DL_{1}-DL_{2})}. \end{array}$$

We can consider the SBM type with the lowest minimal description length most likely; the distance of the posterior odds ratio to 1 determines how much more likely it is than the other SBM type.

We performed 500 runs with random initial partitions for each of the four SBM types and each of the six networks. The SBM type with lowest minimal description length was used for further analyses.

### Overrepresentation analysis

Within the mRNA and protein networks, biological annotation of the blocks and overrepresentation was performed using Reactome pathways and the package ReactomePA ^38^. We restricted our analysis to Reactome pathways containing at least 10 and at most 500 annotated genes, all entities of the network were used as background. We employed Benjamini-Hochberg multiple-testing correction. Pathways were considered overrepresented for default settings (p-value < 0.05, q-value < 0.2), and only human pathways occurring in the file from the Reactome database, https://reactome.org/download/current/ReactomePathwaysRelation.txt (downloaded June 6, 2018, 39), were used. This file was also employed as representation of the Reactome hierarchy to determine distances between Reactome pathways.

For the metabolite dataset, we mapped the pubchem IDs and metabolite names to KEGG compound IDs using the MBRole webserver version 2 ^40^ and merged them semi-manually, thereby preferring metabolite names (in case of mismatch with pubchem record) and KEGG IDs with pathway annotation. We downloaded the human KEGG pathway annotation for the mapped metabolites from MBRole and used it as user-defined annotation for overrepresentation analysis with the *enricher* function from the package clusterProfiler ^41^. We only considered pathways with a minimal size of 2 and otherwise the same settings as for mRNA and protein overrepresentation analysis.

### Comparison to breast cancer signatures and summarizing biological function

For comparison to known biological functions relevant to breast cancer, we downloaded those gene sets from the MSigDB database v6.2 ^42^, gene set collection C6, oncogenic signatures, that arose for the search terms "breast AND (cancer OR carcinoma)" on the MSigDB web interface (17 gene sets in total, search and download on July 31, 2019). We computed the Reactome pathways that are overrepresented in any of these gene sets (as described in section "Overrepresentation analysis"). The resulting 43 Reactome pathways were assorted according to their Reactome parent pathways (top level Reactome pathways) and, for each single pathway, its occurrence among pathways overrepresented in SBM-predicted blocks was counted.

For summarizing biological function, we mapped each overrepresented Reactome pathway to its parent Reactome pathway (top-level pathway). For each network and each hierarchy level, we counted the number of occurrence of each Reactome parent pathway among the Reactome pathways overrepresented in any of the SBM-derived blocks. We normalized these counts to the total number of overrepresented pathways in the blocks of the network and hierarchy level in order to obtain the percentage of each Reactome parent pathway. Note that we counted the same pathway multiple times if it was overrepresented in multiple SBM-derived blocks. In order to account for the different sizes of the Reactome parent pathways, we further divided the percentage of a Reactome parent pathway for a network and hierarchy level as described above by the percentage of all Reactome pathways that are associated to the Reactome parent pathway. This latter percentage is for example high for "metabolism" and "signal transduction" and low for "chromatin organization" and "circadian clock".

### Distance measures of Reactome terms and within hierarchical SBMs

We employed two distance measures of Reactome pathways considering the graph given by the Reactome familial hierarchy tree: (i) the distance in terms of number of steps necessary in the Reactome hierarchy graph to reach from one pathway to the other (distance 1), and (ii) the hierarchy level of the lowest common ancestor, or least common subsumer, for ontology graphs (distance 2). We incorporated an artificial top-Reactome pathway into the hierarchy to connect all pathways with each other and to have our distance measures well-defined. It lies at the 10th hierarchy level, so the largest distance between two pathways is 10 for distance 1 and 18 for distance 2 (nine steps to the highest level and nine back). For all comparisons, only blocks with at least one overrepresented pathway were considered. For comparing the distances of Reactome pathway annotations between blocks, i.e. to associate a distance to a pair of blocks, we median-averaged the distances over all combinations of Reactome pathways associated to the two blocks. For distances of pathway annotations within blocks, we median-averaged the distances between all possible combination of different Reactome pathways associated to the block. The trivial distances of zero for the distance of a Reactome pathway to itself were omitted, as well as blocks with only one overrepresented Reactome pathway for intra-block distances. Distances between blocks in the SBM hierarchy, as employed in Figure 3D, were defined analogously to the distance measure (i) based on the graph of the SBM hierarchy.

### WGCNA clustering

Alternative clustering using the WGCNA package ^3^ was performed following the WGCNA tutorial on clustering. We employed the full correlation matrices including self-edges. First, the correlation values were scaled to values between zero and 1 (by (1+corr)/2), and the soft threshold delivering scale free network topology was determined using the *pickSoftThreshold.fromSimilarity* function with default settings. Power estimates were 8, 16 and 12 for the mRNA, protein and metabolite network, respectively. We calculated the dissimilarity using *TomSimilarity* on the soft thresholded correlation matrix, used *hclust* with method "average" and *cutreeDynamic* with "deepSplit" parameter of 4 (mRNA) or 2 (else), "pamRespectsDendro" set to FALSE and "minClusterSize" of 3 (for mRNA, metabolite) or 4 (for proteins) to make the clustering most similar to the one obtained from the SBM.

### Edge prediction

We aim to derive edge confidence scores for each single edge in the network exploiting the representation of the network as SBM. Let us consider a fixed network given as graph *G*. If *δG* is a set of edges which do or do not occur in the network *G*, the probability that these edges belong to the observed network (for edges missing in *G*) or do not belong to the observed network (for edges from *G*), *P*( *δG*, *G*), can be written as ^27^:

$$P(\delta G|G)\propto \sum_{b}\frac{P(G+\delta G|b)}{P(G|b)}P(b|G)$$

for *b* being partitions of the network *G* (please refer to *Fit to SBM* for further information on notation). The derivation assumes that the original network, *G* and the altered network with edges in *δG* added or removed, *G* + *δG*, has been generated by some SBM type (and all probabilities are conditional on that SBM type), and that the set *δG* has been chosen by some uniform distribution among all possible edges. The proportionality factor between the expressions depends on the network G and the number of edges in *δG*, and thus can be in particular neglected if only comparing edge confidences between single edges of a network. Because we aim to score all edges, and due to the sizes of the networks making computations slow, we refrained from sampling over the posterior distribution of the partitions and instead employed the single-point approximation for edge prediction proposed in 27:

$$P(\delta G|G)\propto \sum_{b}\frac{P(G+\delta G|b)}{P(G|b)}P(b|G)\approx \frac{P(G+\delta G|b^{*})}{P(G|b^{*})}P(b^{*}|G).$$

It resorts to neglecting the summands for all partitions except for the one, b*, which contributes most to the posterior distribution, b* = *max _b_P*( *b*| *G*). In addition, the approximation relies on the assumption that the estimated optimal partition for the representation of *G* by the SBM is the same for *G* and its altered version with added or removed edge, *G* + *δG*, which is reasonable for our application case of single edge predictions, i.e. for *δG* being composed of a single putatively missing or spurious edge. The term *P*( b*|G) can be considered constant for a fixed *G* and SBM type, so we can shift it to the proportionality factor. Considering the microcanonical formulation of the SBM (see *Fit to SBM*), it becomes clear that the edge predictions for *δG* directly depend on the difference between the description length of the original network *G* with partition b*, *DL*_G,b*_, and the description length of the altered network *G* + *δG* with partition b*, *DL*_*G*+ *δG*, b*_^27^:

$$P(\delta G|G)\propto \frac{P(G+\delta G|b^{*})}{P(G|b^{*})}=\frac{P(G+\delta G|b^{*})P(b^{*})}{P(G|b^{*})P(b^{*})}=\frac{2^{-DL_{G+\delta G,b*}}}{2^{-DL_{G,b*}}}=2^{DL_{G,b*}-DL_{G+\delta G,b*}}.$$

The difference between the description lengths, *DL*_*G*, b*_ − *DL*_*G*+ *δG*, b*_, was computed via the function *get_edges_prob* from graph-tool ^36^. Note that as of time of writing, *get_edges_prob* does not work for weighted SBMs with real-normal edge covariate (see filed issue #452 at *graph-tool.skewed.de*). In addition, this function employs the natural (instead of the dual) logarithm and consequently, the obtained value has to be scaled by log _2_( *e*) to obtain the plain difference of description lengths.

In order to make clear that neither these edge predictions nor their dual (or natural) logarithm correspond to actual probabilities, we used the term edge confidence scores or simply edge scores throughout the manuscript.

## Results

### Data preparation, network generation, and reduction

We showcase the SBM-based analysis for data from a subgroup of breast cancer patients: those with estrogen receptor negative (ER-) tumours. ER status is predictive of patient outcome, with ER- leading to unfavourable prognoses, and its assessment is part of standard breast cancer patient screening ^43– 45^. We used three types of molecules reflecting different characteristics of tumorous tissue or cells: mRNAs, proteins or metabolites. These are key cellular molecules that are widely applied, in isolation or in combinations, in different biomedical research domains. Their abundance and interconnectedness in networks are therefore of high interest if aiming to characterize cells or tumorous tissue.

Other potential data types could be e.g. mutations, copy number variation, DNA methylation or miRNAs, which are interesting avenues to further explore. While they could be useful, there are some caveats associated with them, e.g.: the derived interactions within layers are even less directly interpretable than for mRNA, protein or metabolite; networks generated from mutations and copy number variation are extremely sparse; the functional interpretation of DNA methylation data relies on mRNA expression and the resulting networks are extremely big; the roles of miRNAs are less well known. Therefore, we decided to restrict our analyses to the three biomolecular entities mRNA, proteins and metabolites.

The Cancer Genome Atlas (TCGA) initiative has provided data on the mRNA level (from RNAseq) for 237 ER- breast cancer patient tissue samples; mass-spectrometry-based proteomic data (4plex iTRAQ) are available for a subgroup of 36 patients ^29^. Metabolomics data have been measured by GC-TOF-MS in a different breast cancer cohort study for 67 samples ^30^. We treated each measured entity as node and established a correlation-based, weighted, biomolecular network for each single measurement layer. Therein, each pair of nodes is connected by an edge for which the weight is determined by Spearman’s correlation of the measurements of the nodes (over the samples), delivering values between -1 and 1. Thus, an edge that connects nodes with a correlation close to 1 or close to -1 represents strong positive regulation and strong negative regulation, respectively; edges connecting nodes with a correlation close to zero represent weak or absent regulation. We employed Spearman’s correlation because it captures also non-linear relationships between measurements, and it is robust to outliers and any monotonous transformation (e.g. logarithmization). We dealt with missing values in the data by replacing them by small values (only for NAs due to log transformation of zero counts in the mRNA data) or removing entities with >20% missing values for all samples (for the protein data). Subsequently, we computed the correlation only considering pairwise complete observations. The distributions of the computed correlation values for the three data types are shown in Figure 1B.

The resulting biomolecular networks capture the relationships of the intracellular machinery, and their analyses deliver important insights on altered regulations in disease states ^2, 3^. However, because these networks are fully connected, i.e., every entity is connected to each other entity within a layer, the networks become very large and their analyses difficult. A common approach is to reduce the networks, either by selecting a subset of entities as nodes prior to network establishment, mainly by using criteria on abundance, or by using the assumption that weak regulations are less important for the biological network and can be omitted without impairing the represented function of the network. We decided on the latter approach in order not to bias our choice of considered molecular entities and because we wanted to focus on the connections between species, i.e. the covariation of expression. Thereby, also lowly abundant species can exhibit strong connections, and indeed they are found to play a role, as indicated by a non-zero degree, in our reduced networks (see Figure S1D ^46^).

We used two different techniques of network reduction by thresholding: In the first, we only kept edges for which the correlation was significantly different from zero ("sign. corr."), i.e., the regulation being sufficiently strong. Therefore, we computed the p-value associated to each correlation value in the networks. Then, for each of the three networks, we applied both Bonferroni and Benjamini-Hochberg multiple testing correction methods along with the classical significance thresholds 0.01 and 0.05 (see Figure 1C). We finally chose the correction method and significance threshold for each data layer considering a trade-off between minimal network size (i.e. minimal computation time for the subsequent fit to SBM) and maximal connectedness of the reduced network: We used the combination of multiple testing correction and significance threshold that provided a high degree of reduction while maintaining a high percentage of nodes within the largest connected component of the network. While the stringent Bonferroni correction was necessary to achieve a sufficient degree of reduction for the mRNA network, it severely disrupted the connectedness for the protein and metabolite data layer leading to less than 30% or 65% of the nodes being in the largest connected component for protein or metabolite, respectively (see Figure 1C, employed thresholds marked in blue).

In the second reduction method, we systematically removed links weaker than increasingly stringent correlation thresholds until the reduced network met a criterion of scale-freeness (using a function from the WGCNA R package ^3^), see Figure 1D. Scale-freeness is considered a key property of self-organized networks and thus also of biological molecular networks ^1^. In scale-free network formation, highly connected nodes tend to attract more connections than lowly connected nodes leading to a degree distribution following a power-law with a negative exponent. The least stringent correlation threshold for which a sufficiently good fit (scale-free fit index *R* ^2^ > 0.85, 3) between the degree distribution of the reduced network and a power-law degree distribution with negative exponent was obtained was used for network reduction (networks named "scale-free"). The degree distributions of all six reduced networks are shown in Figure 1E. The relationship between the two reduction by thresholding approaches are further illustrated in Figure S1A-C ^46^. Both reduction techniques are hard-thresholding techniques meaning that edges are removed from the networks. The resulting six reduced networks, two for each data layer, were used in a binary form, i.e., weight information was discarded after reduction. Some characteristics of the original and reduced networks are shown in Table 1. In the following sections, we describe how to analyze these different homogeneous cancer networks by fitting them to SBMs.

**Table 1.** Characteristics of the networks derived from the three data layers. Note that genes/proteins/metabolites were removed if having >20% NA values (which was the case only for proteins), all other entities were kept as nodes in the reduced network even if their degree was zero (i.e. having no edge) after network reduction.

Characteristic	mRNA	protein	metabolite
samples	237	36	67
entities (nodes, before NA removal)	18321	10625	162
edges (before reduction)	167820360	56440000	13041
**reduced network: scale-free**			
minimal correlation	0.603	0.788	0.375
entities (nodes)	18321	8430	162
edges	287790	85980	1825
entities of degree zero	8183	4967	4
entities in largest connected component	9111	3187	158
**reduced network: sign. corr.**			
minimal correlation	0.395	0.534	0.310
entities (nodes)	18321	8430	162
edges	4260267	2434159	2811
entities of degree zero	1061	3	1
entities in largest connected component	17190	8427	161

### Fitting the reduced networks to stochastic block models

Biological networks are known to be modular and hierarchical. Different molecular entities, such as genes, mRNAs, and proteins, are interconnected and form different modules to fulfill a specific function. Modularity can convey more robustness to the overall system, e.g. by preventing perturbations in single modules to spread fast and to cause erroneous behavior in other modules and thus functions. Hierarchies capture two characteristics of biological systems: (i) the ordered combination of functions, i.e., multiple simple functions resulting in more complex behavior or responses; and (ii) the inherent levels of complex organization of life, from single molecules to cell organelles, cells, tissue, organs and whole organisms.

**Figure 2.**
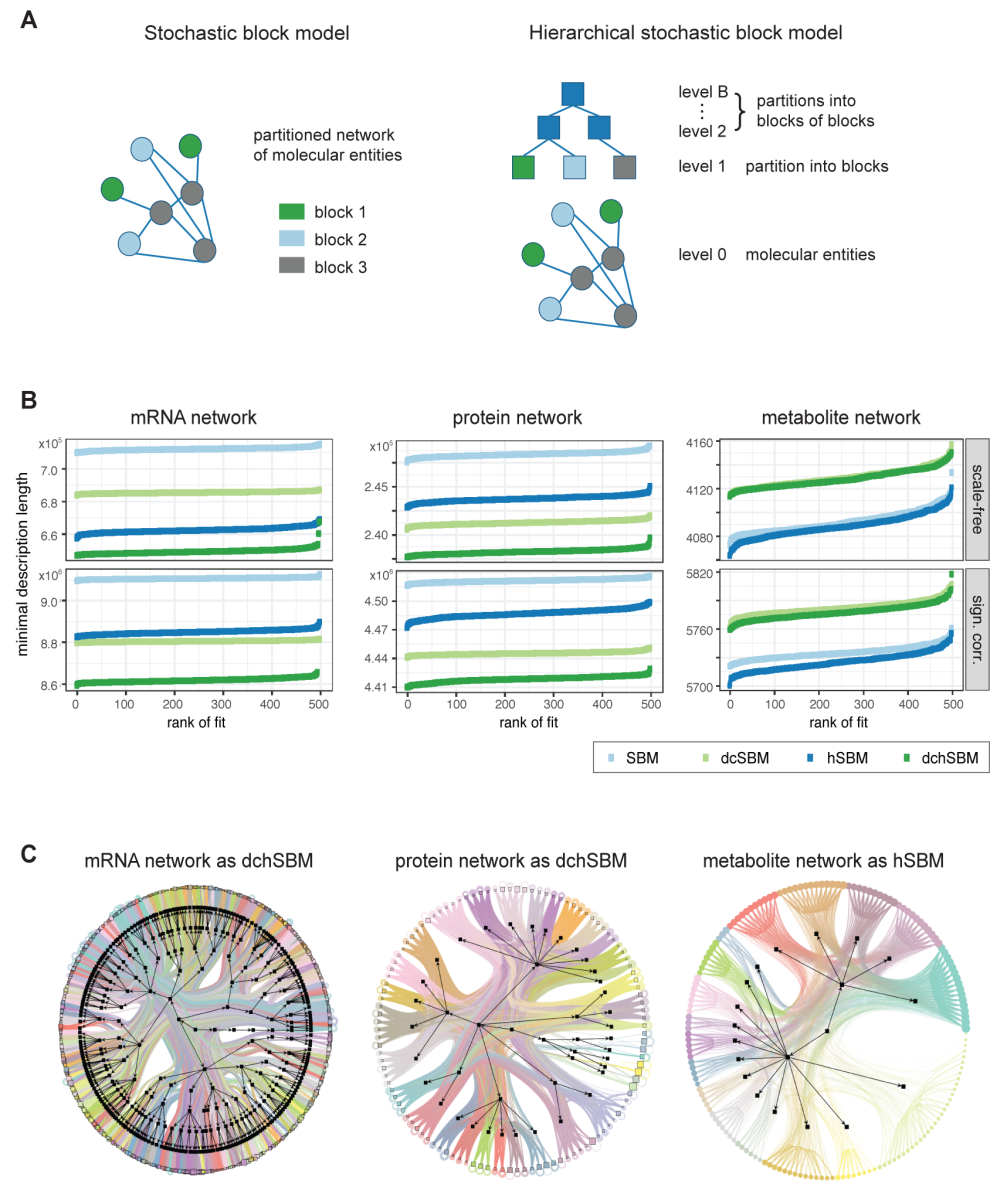
Stochastic block models for representing correlation-based biomolecular networks. ( **A**) In a stochastic block model (SBM) representation, the nodes of a network are partitioned into blocks according to their similarity in connectivity. The hierarchical version of the SBM (right) imposes in addition hierarchical partitions onto the blocks. ( **B**) Six biomolecular networks derived from transcriptomic, proteomic or metabolomics data of breast tumours were fitted to four different types of SBM: the classical SBM (SBM, light blue), the degree-corrected SBM (dcSBM, light green), the hierarchical SBM (hSBM, dark blue) and the degree-corrected hierarchical SBM (dchSBM, dark green). We performed fits for 500 initial partitions for each network. A fit consists of altering the partitions underlying the SBM such as to minimize the description length. The smaller the description length the better the fit. Hierarchical SBMs outperform non-hierarchical SBMs, degree correction is required for the mRNA and protein networks. ( **C**) Graphical representations of the best fitting hierarchical stochastic block models with degree correction (dchSBM) or without (hSBM) for the networks reduced by significance of correlation (mRNAs, metabolites) or by scale-freeness (protein). The lowest layer (genes, proteins) is truncated in the mRNA and protein networks, colored lines denote edges between blocks of the first level (for mRNA, protein network) or between metabolites (level 0, metabolite network). Edges of blocks or metabolites which belong to the same block in the level above have the same colour. The higher-order hierarchical structure is shown in black.

The stochastic block model (SBM) is the simplest form of a generative network model based on communities, i.e., group structures of the nodes. Thereby, nodes are assigned to blocks according to their connectivity properties ( Figure 2A left); the block associations of two nodes fully determine the probability of an edge between them. A shortcoming of the classical SBM for representations of real networks is that nodes within one block need to have similar degrees. The degree-corrected version of the SBM ^35^ accounts for that and enables different degrees for nodes within a block. Another extension of the SBM is its hierarchical version, in which the blocks are further partitioned into blocks of higher levels ^25^ ( Figure 2A right). This model is especially suitable to represent large networks with many nodes as it counteracts underfitting. Fitting biological networks to hierarchical (also called nested) SBMs is therefore most appropriate.

We assessed which of the four following types of stochastic block models could best represent the biological networks: the classical SBM, the degree-corrected SBM, the hierarchical SBM or the degree-corrected and hierarchical SBM ( Figure 2B). We used the Python module graph-tool ^36^ to fit SBMs to the networks, i.e., to find which partition (basal and/or hierarchically ordered) describes the network best as SBM.

Note that we also examined the performance of weighted stochastic block models for our purpose of edge prediction as they have been successfully employed before for non-biomolecular networks ^18, 27, 47^. However, the characteristics of the optimal weighted SBM (number of blocks) were severely impacted by prior assumptions on the edge weight distributions, and the derived edge confidence scores did not coincide well with evidence on edge relevance given by the correlations of the edges for fully-connected weighted networks (see Figure S2 ^46^). Taking in addition the increased computational effort for fitting the weighted SBM compared to the binary version into account, we restricted our further analyses to non-weighted SBMs.

The model fit is performed following the rationale of Occam’s razor: The simplest model describing the data is the best. Thus, we searched for the partition that minimized the description length, i.e., the amount of information necessary to describe the network as an SBM. Additional information required to capture degree-correction and/or hierarchies compared to the classical SBM is thereby taken into account. Consequently, it can be directly concluded which of the four SBM model types is most appropriate for a certain network: The one with the lowest minimal description length. The optimization of the description length runs via an agglomerative Markov Chain Monte Carlo algorithm ^37^. It is non-deterministic and multiple initiations of the underlying partition of the SBM are required to obtain globally instead of locally optimal partitions. We performed optimizations for 500 initial partitions for each network and SBM type.

For all four mRNA and protein networks, the classical SBM delivered the worst fit and the degree-corrected, hierarchical SBM fitted best ( Figure 2B left and middle). Degree correction did not prove necessary to describe the metabolite networks, for which the hierarchical SBMs fitted slightly better than the classical SBM ( Figure 2B right). A graphical representation of the best fitting SBMs in circular layout, showing the blocks from the lowest layer (for mRNA, protein) or the metabolites (metabolite network) and their connections in color, and the hierarchical structure on top in black, is given in Figure 2C.

### SBM-derived communities of the biomolecular networks capture and predict biological function

We wanted to assess how well the biological content of the biomolecular networks is captured in the stochastic block models, i.e., if the clustering of the nodes into the blocks is biologically meaningful. To that purpose, we estimated whether the blocks show common biological function based on Reactome or KEGG pathways. Reactome provides a hierarchical annotation which enables a definition of a distance between terms and a comparison to the hierarchical structure given by the SBM, KEGG is one of the annotation databases used most for metabolites. In particular, we performed overrepresentation analyses of Reactome pathways for the blocks at each level of the SBMs of the four protein and mRNA networks, and overrepresentation of KEGG pathways for the blocks predicted for the two metabolite networks.

**Figure 3.**
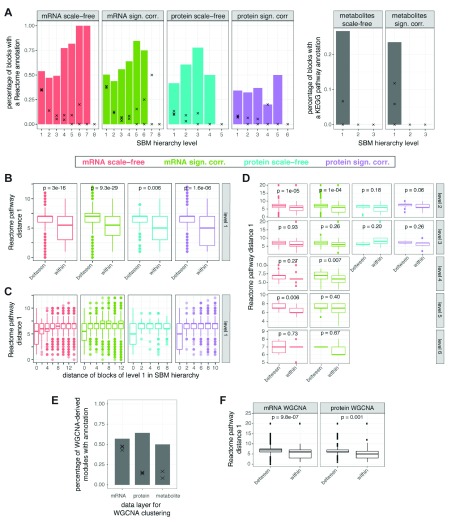
Modules derived from hierarchical SBM represent biological function. ( **A**) Percentage of blocks with at least one overrepresented Reactome (KEGG) pathway for the best fitting SBM (see Figure 2B) for the mRNA and protein (metabolite) networks, reduced by condition on scale-freeness (scale-free) or on significance of correlation (sign. corr.), for each hierarchical level (bars). Black crosses denote the percentages of blocks with at least one overrepresented Reactome (KEGG) pathway for three SBMs each with exactly the same structures but randomly shuffled mRNAs or proteins (metabolites). ( **B**) For the lowest hierarchy level clustering (level 1) of each of the four mRNA and protein SBMs, we calculated the average distances between every pair of Reactome pathways between blocks and those within blocks, for a distance measures (i) based on the Reactome hierarchy (see Figure S3 ^46^ for the results using the alternative distance measure (ii)). The lower the distances the more similar are the pathways. The pathways associated to one single block (within) are significantly more similar than those associated to different blocks (Welch’s t-test p-value < 0.01) suggesting that biological functions are consistent within blocks and distinct between blocks. ( **C**) Distance of Reactome pathways (as in **B**) between blocks (or within blocks, for distance of blocks in SBM being zero) versus the distance of the blocks in the SBM hierarchy for blocks on level 1. We do not find evidence that the Reactome hierarchy is reflected in the SBM hierarchy. ( **D**) Between-module vs. within-module distances of Reactome terms as in ( **B**) for blocks of SBM hierarchy levels 2-6. ( **E**) Percentage of modules detected by the WGCNA approach with at least one overrepresented Reactome or KEGG pathway. Black crosses denote the results for three similar clusterings with randomly shuffled mRNAs, proteins, or metabolites. ( **F**) Between-module vs. within module distances of Reactome terms as in ( **B**) for the clusterings predicted from WGCNA-based module detection.

We find that a high percentage of blocks in each level has at least one associated Reactome or KEGG pathway, i.e., the genes of the pathway are overrepresented within the block, they occur more frequently than expected by chance ( Figure 3A bars). Except for the highest hierarchy levels that consist only of a few blocks, this percentage is decisively higher than for a random clustering of exactly the same structure (results for 3 random clusterings shown as black crosses in Figure 3A).

Many blocks, however, have not only one but multiple Reactome pathways assigned. If blocks are biologically meaningful, we would expect to observe similar Reactome pathways within blocks but less similar pathways when comparing the pathways of different blocks.

The dissimilarity of Reactome pathways is naturally represented by their distance in the Reactome familial hierarchy structure; the more distant pathways are, the less connected are their biological functions. We used two closely related measures to assess it: (i) the distance of two pathways is the length of the shortest path in the hierarchy tree from one pathway to the other, and (ii) the higher the hierarchy level of the lowest common ancestor (least common subsumer) of two pathways, the more distant they are. Please note that we restricted this analysis to the mRNA and protein networks because the KEGG pathways employed for metabolite data are only assorted into a very shallow hierarchy.

We compared the distances of Reactome pathways using the average of the distance measure over pairs of Reactome pathways associated with one block (within blocks) and over pairs of Reactome pathways associated with two different blocks (between blocks) for the lowest hierarchy level blocks (level 1, see Figure 3B for distance measure (i), Figure S3 ^46^ for distance measure (ii)). Thereby, for all four networks and both measures, we observe significantly smaller distances of Reactome pathways within blocks than between blocks (Welch’s test p < 0.01). This suggests that biological function is coherent within and distinct between blocks, thus further enhancing the notion that SBMs represent well the biological function of the networks.

In addition, we compared Reactome pathway distances between blocks and within blocks for the blocks of the higher levels (levels 2-6, Figure 3D). Please note that this analysis could only be performed for the subset of SBM hierarchy levels with at least three blocks with more than one overrepresented Reactome pathway (otherwise, a Welch’s test cannot be performed due to low sample count). For higher levels, within-block distances are only in some cases significantly lower than between-block distances, no general effect can be observed.

Furthermore, we examined the relationship between the hierarchies within the SBMs and the hierarchy of the Reactome pathways. First, we defined the distance of blocks within a hierarchical SBM by counting the number of steps necessary to reach a block from the other (via their lowest common higher-level block). Second, we compared the distance within the SBM hierarchy of each pair of blocks (on level 1) to the distance of their associated Reactome pathways within the Reactome hierarchy ( Figure 3C, Figure S3B). Thereby, we found only a weak positive correlation for Reactome pathway distance measure (i), that is even further reduced if neglecting intra-block coherence, i.e., neglecting blocks with distance zero in the SBM hierarchy. From both analyses ( Figure 3C–D), we concluded that the hierarchy within the SBM does not strongly coincide with the hierarchy within the Reactome pathways.

In order to assess how the clustering by SBM relates to established clustering techniques in correlation-based networks, we also performed module detection by using the WGCNA package ^3^. Note that for this approach, no model reduction is necessary, which means that we obtain only one result for each data layer. After soft thresholding to enforce scale-free network architecture, we employed WGCNA module detection to obtain comparable numbers of modules as for the SBM-based approach: 333 (mRNA), 109 (protein) and 12 (metabolite) for WGCNA; these numbers are similar to the 438, 113 and 15 blocks detected by SBM for the corresponding scale free networks. Overall, the WGCNA clusterings show a larger diversity of module sizes than those obtained for the SBM approach (Figure S3D ^46^). For all three data layers, a higher percentage of WGCNA-derived modules have a biological annotation compared to the blocks from the SBM (compare Figure 3E to 3A). However, for the mRNA layer, many of the blocks also have an overrepresented pathway annotation for randomly shuffled gene names which hints on reduced significance of the WGCNA results and better performance for the modules detected by fitting to SBM. For protein and metabolites, in terms of detection of biologically annotated modules, the WGCNA approach seems to provide slightly better results than the SBM approach. Comparison of within-module distances and between-module distances of the annotated Reactome terms for the mRNA and protein networks delivers significantly lower within-module distances as for the SBM clusterings ( Figure 3F).

The observed differences derived from WGCNA vs. SBM could stem from conceptual differences in the approaches: Instead of detecting clusters of entities with highly positively correlated abundances only as in WGCNA, nodes from the same block are characterized by common connectivity characteristics in the SBM. Clearly, as proteins interact and bind directly for complex formation or regulation, and metabolites are interconverted into one another, entities which act together tend to have similar abundances and thus the modularization by WGCNA shows good results. For the detection of modules for mRNAs whose interaction can be considered less direct, assorting entities with similar connectivity patterns as in the SBM-derived modules is beneficial. In addition, the WGCNA framework, as most other module detection methods, cannot aid in assessing edge relevance - which is enabled by the fit of the networks to SBMs.

**Figure 4.**
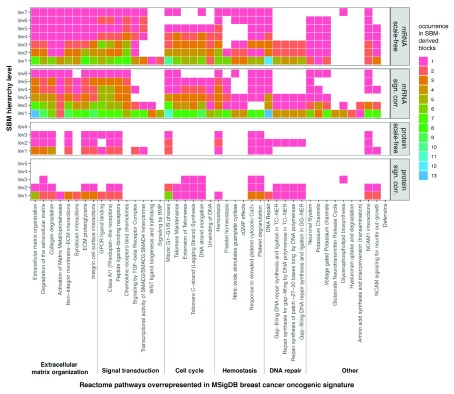
SBM-derived blocks of mRNA and protein networks exhibit biological functions related to breast cancer signature. Oncogenic signature gene sets related to breast cancer were retrieved from MSigDB ^42^ and mapped to Reactome pathways. The color code shows for how many SBM-derived blocks each of these pathways was overrepresented, for the four mRNA and protein networks and each hierarchy level. Hierarchy levels without any blocks with overrepresented pathways are omitted.

We furthermore compared the results from the overrepresentation analysis of the SBM-derived blocks to known biological insights from breast cancer. To that purpose, for the mRNA and protein networks, we compared the SBM-derived biological Reactome terms to those found for oncogenic signatures obtained from the database MSigDB ^42^. The 43 Reactome pathways known to be related to breast cancer according to MSigDB (see *Methods*) stem mainly from the categories extracellular matrix (ECM) organization, signal transduction, cell cycle, hemostasis, DNA repair, and few others. All but one of these pathways were found as overrepresented, partially with high frequency, in SBM-derived blocks of one of the mRNA or protein networks ( Figure 4). The exception is "Defensins" that are relevant for antimicrobial immune response and therefore the immune system. Its occurrence in oncogenic signatures might originate from immune cells measured together with tumour tissue. Overall, the two protein networks exhibit less overrepresented pathways, but especially the categories ECM organization and DNA repair are well represented in both networks, and cell cycle in the network reduced by significance of correlation (lower 2 panels in Figure 4). Thus, biological functions related to breast cancer are well captured in the SBM-derived clustering.

For the metabolite networks, we investigated the KEGG pathways found as overrepresented in the SBM-derived blocks ( Table 2). Therein, especially "Biosynthesis of unsaturated fatty acids" is very prominent, it is indeed overrepresented in two blocks for each network (not shown) and occurs in different variations in the scale-free network (e.g. further overrepresented terms "Fatty acid biosynthesis", "Linoleic acid metabolism", Table 2). Indeed, fatty acids synthesis has been related to metastasis, therapeutic resistance and relapse in cancer ^48^. The SBM-derived predicted importance of valine, leucine and isoleucine metabolism for breast cancer ( Table 2) has been suggested before, in particular with respect to leucine ^49^. In addition, ABC transporters occur as overrepresented in both SBM-derived clusterings of the metabolite networks: They have been suggested to play a role in chemoresistance ^50^ and therefore point to one possible underlying reason of the bad prognosis of ER- breast cancer patients.

**Table 2.** Overrepresented KEGG pathways for the SBM-derived blocks of the metabolite networks (reduced by significance of correlation, sign. corr., or by a criterion on scale-freeness, scale-free).

metabolite network: sign. corr.	metabolite network: scale-free
Biosynthesis of unsaturated fatty acids	Biosynthesis of unsaturated fatty acids
Aminoacyl-tRNA biosynthesis	Aminoacyl-tRNA biosynthesis
ABC transporters	ABC transporters
Valine, leucine and isoleucine biosynthesis	Valine, leucine and isoleucine biosynthesis
Valine, leucine and isoleucine degradation	Valine, leucine and isoleucine degradation
Amino sugar and nucleotide sugar metabolism	Starch and sucrose metabolism
	Fatty acid biosynthesis
	Linoleic acid metabolism
	Ubiquinone and other terpenoid-quinone biosynthesis
	Biosynthesis of secondary metabolites
	Glycine, serine and threonine metabolism

**Figure 5.**
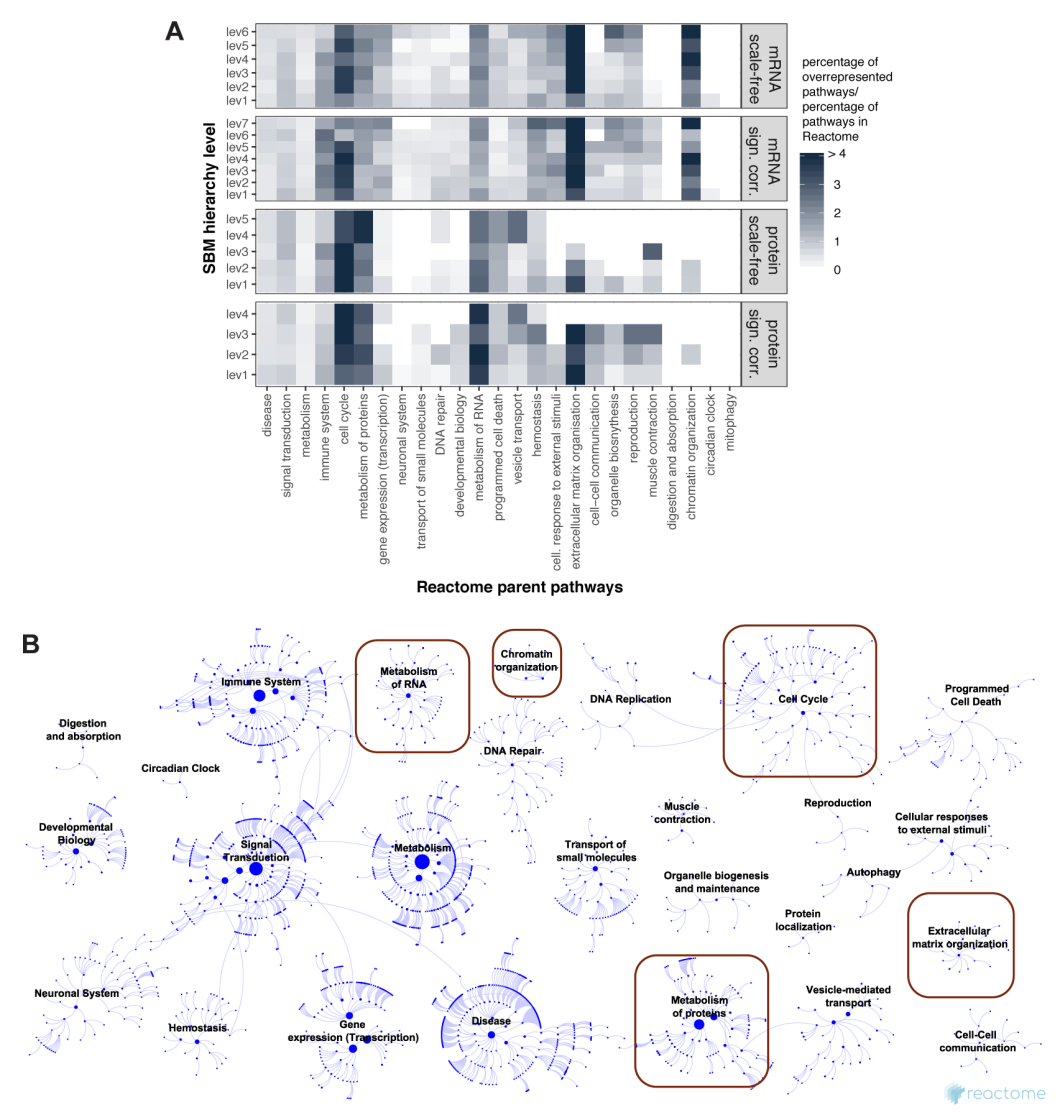
SBM-derived blocks of mRNA and protein networks predict further pathways relevant for breast cancer. ( **A**) We mapped the Reactome pathways to their Reactome parent pathways and counted their occurrence as overrepresented pathway in SBM-derived blocks of the four mRNA and protein networks and each hierarchy level. Reactome parent pathways are sorted by the number of pathways they summarize (see ( **B**)). The color code gives the percentage of occurrence as overrepresented of each parent pathway (counted occurrence divided by total number of overrepresented pathways obtained for the SBM-clustering of the hierarchy level and network) relative to the percentage of each parent pathway in the Reactome annotation (number of pathways associated to the parent pathway relative to the number of total Reactome pathways). These values indicate whether certain parent pathways occur more frequently than suggested by the size of the parent pathways cluster in Reactome. The higher the value (i.e. the darker the color), the more relevant the parent pathway is predicted by the SBM-derived network structure. ( **B**) Reactome pathway organization (downloaded from the Reactome website Pathway Viewer, 39). The parent pathways that are predicted as especially relevant for breast cancer according to ( **A**) are highlighted.

In order to identify further biological functions that could play a role according to SBM-derived network structures in mRNA and protein networks, we summarized the overrepresented Reactome pathways on the level of the parent Reactome pathways (on the top level of the Reactome hierarchy, Figure 5). Thereby, we could retrieve the categories known to be of relevance to breast cancer, in particular ECM organization and cell cycle, for all four networks. We found additional categories that were observed more frequently than they are represented in the Reactome hierarchy: "Metabolism of RNA", "Metabolism of proteins", and "Chromatin organization" (columns with darker color in Figure 5A). Please note that tRNA synthesis has occurred as overrepresented KEGG pathway of the SBM-derived structures of the metabolite networks ( Table 2) - since tRNA is subject to RNA metabolism and is required for the translation of proteins, it lies at the interface those two relevant metabolism categories predicted from mRNA and protein networks and thus complements their predictions. Metabolism of proteins relies on amino acids and could be also related to the leucine addiction reported for breast cancer ^49^, and it is supported by the SBM-derived predictions in the metabolite network on the relevance of the metabolism of further amino acids ( Table 2). Of note, metabolism in general did not occur with a high frequency (relative to the size of the category “Metabolism”, see Figure 5B) revealing a certain specificity of the predictions obtained by fitting to SBM. The highlight on chromatin organization is an interesting SBM-derived prediction complementary to the metabolism motif and invites further exploration, e.g. by examining related blocks and their connectivity structure in relation to other blocks in the SBM.

To summarize, biological functions related to breast cancer are well captured in the SBM-derived clustering. Networks derived from different datalayers enable different perspectives of the phenotype that can support each other or provide complementary predictions. After the assessment of the biological content in the SBM-derived clustering, we moved to the second deliverable of the fit of the networks to SBMs: finding alternative edge relevance based on global network properties.

### Assessing edge relevance by SBM-based edge confidence scores

The descriptions of the biomolecular networks as SBMs were exploited to determine a confidence score for each existing or non-existing edge of the network. This score would capture how well the existence (or non-existence) of the edge fits to the network’s description by the SBM: Erroneously kept or removed edges lead to a worse fit of the SBM to the network, and removing or reinstalling these edges lead to fit improvement. Indeed, under certain assumptions (see *Methods*), the derived edge confidence score is proportional to the actual absolute probability that the edge belongs to the network, and thus signifies relative edge relevance. Therefore, the confidence score could be used to predict whether an edge is spurious (or missing) ^17, 18, 27^. A high missing edge confidence score suggests that the edge in question is missing and should be restored, it has a high relevance; a high spurious edge confidence score suggests that the edge in question is spurious and should be removed from the network, it has a low relevance. The SBM-based edge confidence scores rely on global network connectivity characteristics and complement the correlation-based weights of the edges which stem from the local, measured characteristics of their nodes.

**Figure 6.**
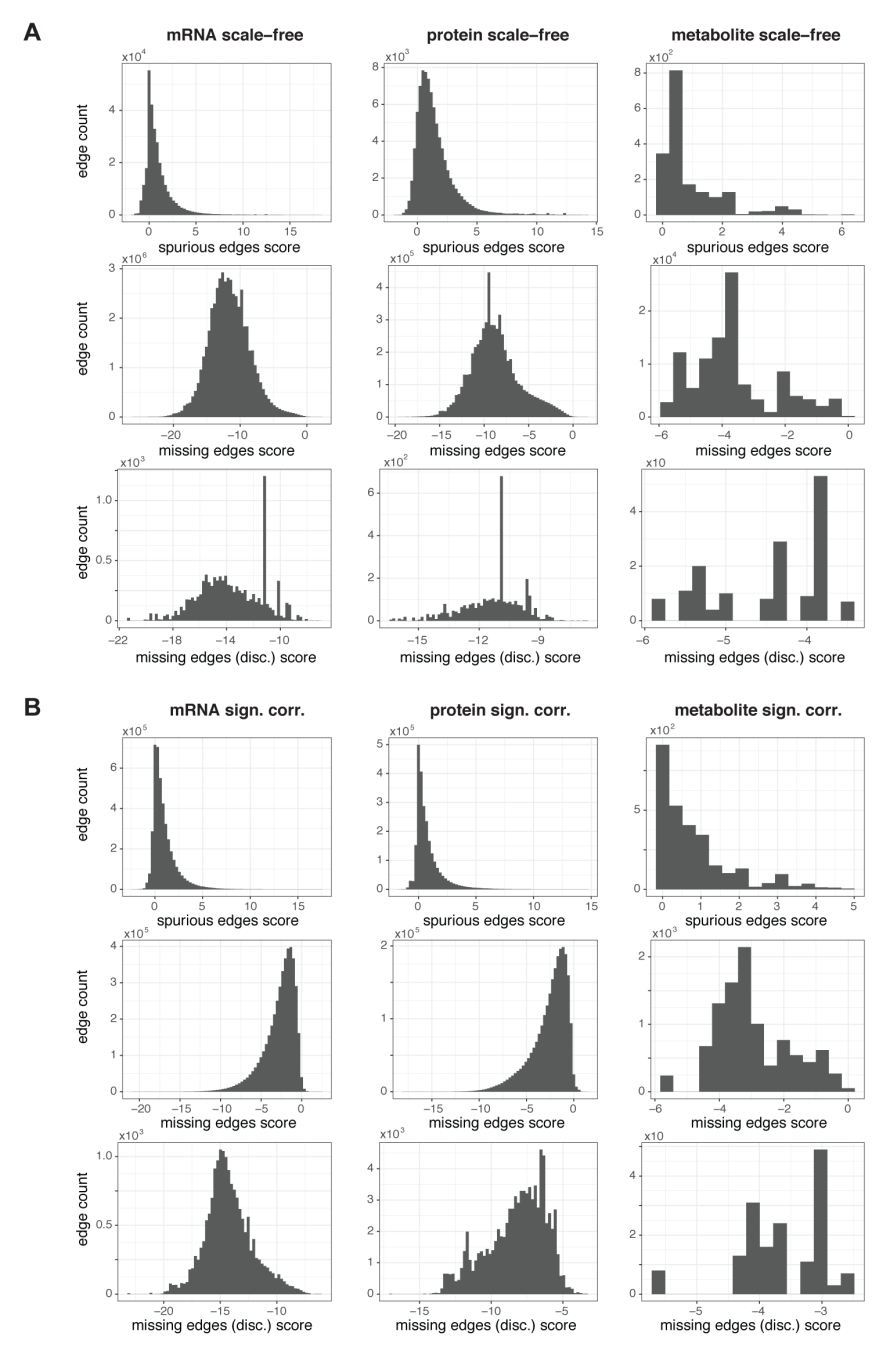
SBM-derived edge prediction: Missing and spurious edge confidence scores. ( **A**) Histograms of edge confidence scores for the best fitting SBM of the three networks reduced by criterion on scale-freeness for all existing, i.e., putatively spurious edges (spurious edge score, top), for the putatively missing edges between nodes with degree > 0 (missing edge score, middle), for the putatively missing edges connecting a node of degree 0 to a node with a larger degree (missing edge (disc) score, bottom - only one edge for each node with degree > 0 is shown). ( **B**) Edge confidence scores as described in ( **A**) for the networks reduced by criterion on significance of correlation. For the mRNA and protein network, missing edge scores (middle) were only computed for the edges with largest absolute correlations of the node measurements.

For the six reduced networks, all edges that existed in the network were considered as "putatively spurious". Similarly, all edges that were not in the network because they had been removed from the (fully connected) correlation-based network during the reduction procedure were considered as "putatively missing". For each putatively missing and spurious edge, we used the Python module graph-tool ^36^ to compute its edge confidence score ( Figure 6). Thereby, we took advantage of the following fact: Entities of degree zero, i.e., nodes that have no connection to any other node in the network after reduction, are indistinguishable to the SBM. Consequently, also all putatively missing edges connecting any of these nodes to a specific second node are only distinguishable by this second node, and thus carry the same missing edge confidence score. This reduces the number of scores we need to compute considerably, depending on the degree of reduction (see counts of entities of degree zero in Table 1). We display the scores for the different types of missing edges in different histograms ( Figure 6A, B middle and bottom). For very big networks with a low degree of reduction (the mRNA and protein networks reduced by significance of correlation), it is still computationally not feasible to compute the scores for all putatively missing edges. We therefore resorted to computing it for as many putatively missing edges as we have existing edges in the network (i.e. approx. 2.4×10 ^6^ for the protein network, 4.6×10 ^6^ for the mRNA network, see Table 1), and chose those with highest absolute edge weights ( Figure 6B left and middle).

Recall that the edge confidence scores are relative, i.e., they serve for comparing relevance between edges only. In addition, computation of the scores relies on the assumption that the partition of the originally fitted SBM is correct for the network. Different edge confidence scores might be obtained for SBMs with different partitions but with similarly good fit to the network. We neglect this complication for the sake of computational efficiency. Still, we have to keep both facts in mind for the interpretation and usage of the edge confidence scores. For example, an evident threshold for declaring an edge as relevant ("missing") or not relevant ("spurious") would be to have the respective edge confidence score larger than zero, as this indicates an improvement in fit quality if adding or removing the edge, respectively. However, we observe an imbalance of our computed scores which is inherent to the approach: Because having less edges reduces the complexity of the underlying network, removing edges preferentially reduces also the amount of information required to describe the SBM, i.e., its description length turns smaller, its goodness of fit improves. Therefore, the edge confidence score distributions of missing edges are shifted to the left - the great majority of edges would be predicted as not missing for the threshold of zero, they are not relevant and should be left out ( Figure 6A, B, 2nd line). Conversely, the spurious edge confidence score distributions are shifted to the right - the great majority of existing edges would be predicted as spurious, they are not relevant and should be removed for an edge score threshold of zero ( Figure 6A, B, top). Both measures point to making the networks smaller.

### Evaluation of edge confidence scores based on correlation

In order to determine whether the edge confidence scores are overall a reasonable assessment of edge relevance, we compared the predicted scores to the edge weights of the edges as derived directly from Spearman’s correlation of the measurements of the nodes. It is important to note that these edge weights (correlations) were used exclusively for the reduction of the correlation-based networks. The edge correlations were at no point provided to the SBM, neither during the fitting to the SBM (except for the weighted version and only for Figures S2, S4 ^46^) nor during the computation of the edge scores. Thus, they are close to an independent validation of the edge relevance, edges with large correlation being more relevant to the system encoded by the network than edges with a correlation close to zero.

**Figure 7.**
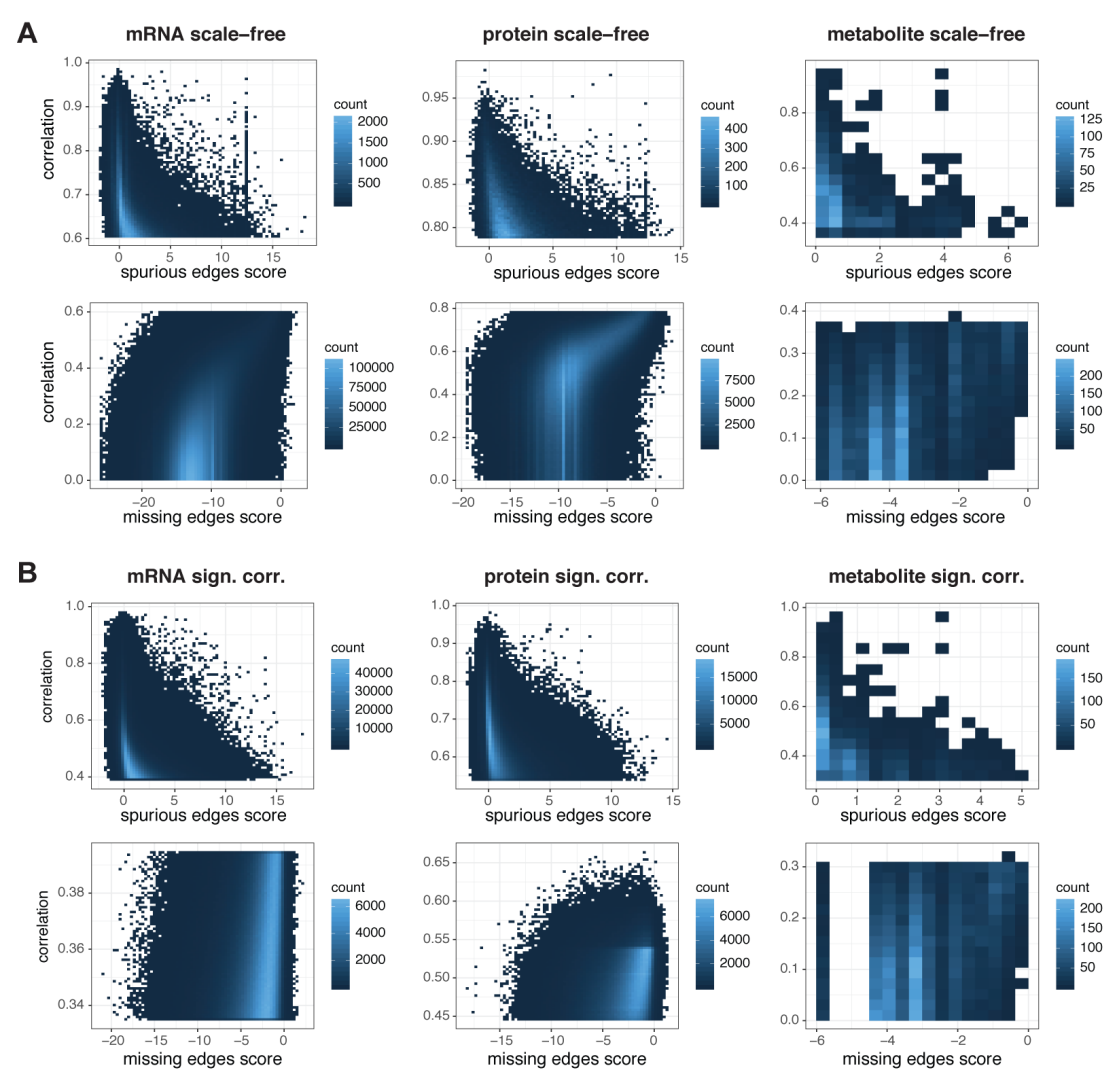
SBM-derived edge prediction: Validation by correlation. Relationship between edge confidence scores and edge correlations (Spearman’s correlation of the measurements at their nodes) for putatively spurious (top) or putatively missing edges between nodes of degree > 0 (bottom) for the networks reduced by criterion on scale-freeness ( **A**) or significance of correlation ( **B**). Edge confidence scores correlate well to edge correlations, with edges with high SBM-predicted confidence in spuriousness - edges predicted as irrelevant - having lower correlations, edges with high SBM-predicted confidence in missingness - edges predicted as relevant - having higher correlations. Please note that for the protein network reduced by significance of correlation, due to missing values in the dataset, a fixed significance threshold leads to different correlation thresholds and thus the correlation value boundary is blurred ( **B**, middle).

Indeed, for all six networks and SBMs, we find an overall positive correlation between the (absolute) edge correlations and the missing edge confidence scores: Edges with high correlation are preferentially predicted as missing, i.e., relevant to the network, also in terms of edge confidence score ( Figure 7). Similarly, we find an overall negative correlation between (absolute) edge correlations and spurious edge confidence scores: Edges with high correlation are preferentially *not* predicted as spurious, i.e., they are predicted as relevant to the network, also in terms of edge confidence score. Consequently, the comparison to the edge correlation suggests that SBM-derived edge confidence scores could be used as additional information for assessing the relevance of edges for multiple omics correlation-based networks.

## Discussion

Using example cases of correlation-based transcriptomic, proteomic and metabolomics networks from breast cancer tumour samples, here we show that and how stochastic block models can be employed for the analysis of biomolecular networks. The networks can be best represented by the hierarchical version of the stochastic block models. This gives rise to biologically meaningful separation of the biomolecules into many functionally relevant blocks. Biological functions related to breast cancer are well captured in the SBM-derived clusters and the networks derived from the different data layers shed light on different perspectives of the phenotype that can support each other and result in complementary predictions. In addition, the SBM framework enables the computation of edge confidence scores that can be used to predict missing or spurious links.

The representation of the networks by SBMs poses a challenge: The model fit and the derivation of edge confidence scores can be computationally very demanding, especially for networks with many edges. This was the case here for the mRNA networks and the protein network reduced by significance of correlation. Therefore, the approach of network analysis by SBM seems most suitable for smaller and/or sparser networks. On the other hand, it delivers two opportunities.

First, modules derived from blocks in SBMs are not only defined as clusters of tight relationships, i.e. co-expression clusters, as obtained with other clustering approaches ^3, 9, 14^. In an SBM, nodes from the same block are characterized by common connectivity characteristics, i.e., common interaction profiles with nodes from the same and from other blocks. Consequently, comparing SBM-derived modules between conditions naturally points towards detecting altered regulations. Indeed, we found our SBM-derived blocks to be biologically relevant to our examined phenotype, and comparing SBM-derived structures between different phenotypes is an intriguing next step.

Second, the derived edge predictions can reproduce the interaction strengths as estimated from measurements. SBM-based edge confidences tend to score missing edges higher that have high correlation, i.e., that would be considered a strong regulation, an important edge. Analogously, existing edges in the network with low correlation tend to be predicted as spurious with high confidence, i.e., as dispensable. Keep in mind that the correlations corresponding to the edges were not provided at any point to the SBM construction. Thus, the SBM approach proves very strong in delivering relevant edge predictions. A further biological validation of the predicted edge confidence scores, for example by comparison to interaction databases, would be an interesting next step.

Still, a question remains: How should the edge confidence scores be translated into edge predictions to alter the network, i.e. to actually remove or re-install edges? There is a natural threshold for declaring an edge as missing or spurious, namely if the respective confidence score would be larger than zero. However, due to the minimal description length approach for SBM-based edge relevance assessment, the confidence scores are shifted towards reducing the networks as much as possible ( Figure 6), such that this natural threshold for deriving the prediction of actual missingness or spuriousness from the confidence scores is not valid. Further examinations on other possible thresholds are required. However, for relative comparisons of relevance between edges in a network the SBM-based scores are suitable.

Additional directions of SBM-based analysis of biomolecular networks remain to be explored.

(i) For edge relevance assessment in other network types, it has been proposed that edge predictions with SBMs are more reliable if resorting to an ensemble of good fits instead of using the best fit only ^27^. Due to the sizes of the employed correlation-based biomolecular networks, this approach is computationally not of practical relevance here, but it would be an interesting point to assess in the future.

(ii) We considered the weighted version of the SBM that could be an interesting option for the analysis of biomolecular networks because it enables representing fully-connected weighted networks as SBM without prior reduction. It could prove exciting especially for smaller networks, e.g. primary metabolites, or in more targeted data analysis approaches. However, in our case, we found hints that the weighted version of the SBM might not be appropriate for the task of edge prediction from SBM-based confidence scores for fully-connected networks (see Figure S2 ^46^). For reduced networks, the results for weighted SBMs seem more promising (see Figure S4 ^46^). A final assessment on the usefulness of the weighted version of the SBMs is still pending, as long as nothing is known about interaction strength distributions between and within modules: The fitted SBM strongly depends on the prior assumptions chosen for these distributions. An extensive analysis of different choices would be required, which is beyond the scope of this study.

We propose to employ SBM-based modules and edge confidence scores as additional pieces of information to make the results of follow-up analyses more robust that rely on relationships between nodes, i.e., on edges and their strength. Edges and their strength play a key role in interpreting biomolecular effects, e.g. of mutations or drugs. If a drug “activates a protein”, this typically means alteration of the protein’s interaction strengths with other proteins or the DNA. The “mutation of a gene” may severely alter the binding properties of the corresponding translated protein to interaction partners. In sum, interactions and their strengths are at the heart of biologically relevant alterations in biomolecular networks, and characterizing the former is required in order to understand the effects of the latter. The SBM framework enables assessing the edge relevance or interaction strength on the basis of consistent, global network characteristics, instead of on the basis of correlation of measurements. Especially for personalized analyses which generally rely on a characterization by only few error-prone measurements of each molecule, this will be crucial to derive more reliable predictions.

## Data availability

### Underlying data

In this study, data from TCGA and CPTAC were used. Proteomics data stem from 29, metabolomics data stem from 30. The metabolomics raw data can only be obtained upon accessing the cited article ^30^; processed data (Spearman’s correlations and associated p-values) can be found in the gitlab repository below.

Code used to perform the analyses together with a detailed work-flow documentation: https://gitlab.com/biomodlih/sbm-for-correlation-based-networks

Archived code as at time of publication: https://doi.org/10.5281/zenodo.3363060 ^46^

License: GNU GPLv3 license.

### Extended data

Zenodo: Analysis of correlation-based biomolecular networks from different omics data by fitting stochastic block models. https://doi.org/10.5281/zenodo.3363060 ^46^. This project contains the following extended data files:

- Figure S1: The relationship between network reduction by significance of correlation or by scale-freeness
- Figure S2: The weighted SBM seems not appropriate for edge prediction from edge confidence scores for fully connected networks
- Figure S3: Pathway characteristics for alternative distance measure, and block size distributions
- Figure S4: Edge predictions for a reduced weighted network with planar or hierarchical weighted SBMs

Data are available under the terms of the Creative Commons Attribution 4.0 International license (CC-BY 4.0).
