# Supplementary material for: Analysis of correlation-based biomolecular networks from different omics data by fitting stochastic block models
Source: F1000Res. 2019 Aug 27;8:465. Originally published 2019 Apr 14. [Version 2] doi: 10.12688/f1000research.18705.2 (PMC6743255; doi:10.12688/f1000research.18705.2)
Supplement: Peer review history

# Peer review: Reviewer response for version 2

**DOI:** 10.5256/f1000research.22342.r52997

**Published:** 2019-09-12

**Authors:** Ana Conesa, Manuel Ugidos

**Affiliations:** 1 Department of Microbiology and Cell Science, Institute of Food and Agricultural Sciences (IFAS), University of Florida, Gainesville, FL, USA; 2 Institute of Biomedicine of Valencia (IBV), CSIC, Valencia, Spain

Revised manuscript satisfied most of the previous comments.

1. **Data preprocessing: **
  Datasets are well characterize by the number of samples and features, the missing data imputation of the three datasets is clarified.
2. **Network generation and reduction: **
  An explanation is included about the use of 10 ^7 ^as a threshold for the number of edges and its relation with the test-multiple correction rates performed. Furthermore, the scale-free reduction is better explained.
3. **Stochastic block models:**
  On the one hand, the SBM model definition is more extensive and the underfitting problem is addressed. On the other hand, although the comment about the number of partitions is correctly answered, this explanation is not included in the body text.
4. **Functional enrichment:**
  The use of Reactome and KEGG is now justified. The description of Figure 3 has improved and a new panel was added. Moreover, two new Figures were included dealing with the biological insights derived from the SBM blocks. Finally, the edge confident scores section is explained in more detail.

 

**New comments about revised manuscript:**

 Figure 3C and 3D both suggest that SBM block hierarchy does not relates with Reactome’s hierarchy. As mentioned in text, SBM blocks are characterized by common connectivity characteristics which may not be completely related with traditional classification based on functions. This is the key point of SBM derived blocks and it is not fully evaluated in terms of biological meaning, i.e, which pathways are more related to the SBM blocks? Also, a figure with the SBM networks (network topology) as in Figure 2C but including tags with enriched pathways should be included in order to evaluate the biological information that could be extracted from the SBM network. In this sense, Figures 4 and 5 are more suitable than Figure 3 as a “validation” of the capability of SBM-derived communities to capture biological function related to breast cancer.

 The validation step previously proposed: “A good approach could be to select a gene X and see if the rest of the genes of the pathways, where gene X is annotated, are in the same block of gene X (or in near blocks in the hierarchy). And repeat this for a high number of genes.” has not been neither performed or considered in the response. If not suitable or possible, please explain why.

We confirm that we have read this submission and believe that we have an appropriate level of expertise to confirm that it is of an acceptable scientific standard, however we have significant reservations, as outlined above.

# Peer review: Reviewer response for version 2

**DOI:** 10.5256/f1000research.22342.r52998

**Published:** 2019-09-09

**Authors:** Lei Xie, Yue Qiu

**Affiliations:** 1 City University of New York, New York, NY, USA; 2 Department of Computer Science, Hunter College, The City University of New York, New York, NY, USA

Authors have addressed the issues raised. No further comments to make

We confirm that we have read this submission and believe that we have an appropriate level of expertise to confirm that it is of an acceptable scientific standard.

# Peer review: Reviewer response for version 1

**DOI:** 10.5256/f1000research.20484.r47834

**Published:** 2019-06-05

**Authors:** Ana Conesa, Manuel Ugidos

**Affiliations:** 1 Department of Microbiology and Cell Science, Institute of Food and Agricultural Sciences (IFAS), University of Florida, Gainesville, FL, USA; 2 Institute of Biomedicine of Valencia (IBV), CSIC, Valencia, Spain

This manuscript describes the application of stochastic block models (SBMs) as a method to construct biological networks from different omics data. Starting from correlation-based networks of breast cancer data, SBM delivers different modules (or blocks) of the network. In order to assess the usefulness of this approach, the authors evaluate the biological meaning of the modules obtained performing a functional enrichment analysis for each module of the network. Finally, the authors include the edge confidence score computation for each edge in the resulting network.

 This novel application of SBMs with omics data is interesting but its utility is not clearly explained in the manuscript. First, the title is confusing, since I understood a single network is obtained from different omics data, i.e. a multiomic integration analysis, however it is not performed. Furthermore, why are these three datasets used? It seems the authors want to evaluate the SBM-based networks starting from datasets with different complexity (different number of features/samples), but the reason is not properly explained.

 As for the SBM model definition, because of the microcanonical formulation because of its hard constraints, network underfitting could be an important issue. Although underfitting is solved using nested (hierarchical) or degree-corrected SBMs, an explanation of this phenomena and how to deal with it would be suitable in “Fit to SBM” methodological section.

 Regarding the functional enrichment, the choice of the pathway database is not clear. Why is Reactome chosen for genes annotation and KEGG for metabolites? Taking into account that breast cancer related biological findings is not the scope of this manuscript, a deeper analysis of biological information revealed by network blocks is necessary: which pathways are block-specific? Which pathway or pathways are present in the bigger block?  Do the pathway distances between blocks have any relation with previous knowledge about breast cancer? Furthermore, the pathway distances within and between blocks are computed just for the lower level of blocks. Since the network is hierarchical, pathway distances can be obtained for each level. This would indicate if the hierarchy is biological meaningful too. Moreover, the same genes are known to be annotated to related pathways. Therefore, this expected result does not represent a validation to determine the biological meaning of the blocks in the network. A good approach could be to select a gene X and see if the rest of the genes of the pathways, where gene X is annotated, are in the same block of gene X (or in near blocks in the hierarchy). And repeat this for a high number of genes.

 Finally, the edge confidence score looks like important to improve the network characterization, however they do not use this score to optimize the final network. I was expecting the comparison between the original SBM-based network and the corrected SBM-based network via edge confidence score optimization. In order to use the edge confidence scores, a way to compute thresholds for missing and spurious edges should be proposed.

 Resuming, SBM-based biological networks are a new way to represent omics data and it represents a novel approach for SBMs. However, a novel application should be coupled with a suitable interpretation of its results. Moreover, the main conclusion or this manuscript is not clear and the main question for a new methodological development is unanswered, why should I use this method?

**Specific points:**

**1) Data preprocessing:**

- Why are the three datasets used? Is it just a matter of different complexity? Datasets are not completely characterized as the number of features is not indicated.
- Authors say missing values of metabolite data were imputed previously. It is not clear whether they performed the imputation or not. In case they did, the algorithm used is not indicated. If missing values of metabolite data were imputed, why weren't the missing values of protein data?

**2) Network generation and reduction:**

- They use different test-multiple correction rates for mRNA, protein and metabolites in order to obtain a similar degree of reduction. The authors should demonstrate this does not lead to any biases. The reduction objective is set at 10 ^7^ edges, is there any reason?
- In Figure 1.D, a linear relationship is expected for the blue dots but it not perfectly linear, why? Could the scale-free reduction be modified to improve it?

 

**3) Stochastic block models:**

- How are the number of initial partitions (500) defined? Is there any relation between the suitable number of initial partitions and the network complexity (size)?

 

**4) Functional enrichment:**

- They use Reactome database for genes annotation and KEGG for metabolites, but there is no an explanation of why that choice.
- Figure 3.C is confusing. I do not understand the objective of this analysis. In order to demonstrate the biological meaning of the hierarchical SBM, the pathway distances comparison within and between blocks at every hierarchical level (as in Figure 3.B) would be better than the provided analysis.
- As mentioned before, a deeper biological interpretation of the network is necessary.
- I am not sure what the clustering analysis is contributing to. Modules obtained by WGCNA shows good results looking at the number significant of pathways, but are they biologically relevant? Are they related to breast cancer?

We confirm that we have read this submission and believe that we have an appropriate level of expertise to state that we do not consider it to be of an acceptable scientific standard, for reasons outlined above.

# Response

**Published:** 2019-08-12

**Authors:** Katharina Baum

**Affiliations:** Max Delbrück Center for Molecular Medicine in the Helmholtz Association, Germany

We thank Dr. Ana Conesa and Manuel Ugidos for their time and effort they invested into commenting our work. They raised important points that we carefully addressed in our revised version 2 of the manuscript. Please find our specific replies and the description of introduced changes below. 

 >This manuscript describes the application of stochastic block models (SBMs) as a method to construct biological networks from different omics data. Starting from correlation-based networks of breast cancer data, SBM delivers different modules (or blocks) of the network. In order to assess the usefulness of this approach, the authors evaluate the biological meaning of the modules obtained performing a functional enrichment analysis for each module of the network. Finally, the authors include the edge confidence score computation for each edge in the resulting network.

 >This novel application of SBMs with omics data is interesting but its utility is not clearly explained in the manuscript. First, the title is confusing, since I understood a single network is obtained from different omics data, i.e. a multiomic integration analysis, however it is not performed.

 >Furthermore, why are these three datasets used? It seems the authors want to evaluate the SBM-based networks starting from datasets with different complexity (different number of features/samples), but the reason is not properly explained.

 >As for the SBM model definition, because of the microcanonical formulation because of its hard constraints, network underfitting could be an important issue. Although underfitting is solved using nested (hierarchical) or degree-corrected SBMs, an explanation of this phenomena and how to deal with it would be suitable in ‘Fit to SBM’ methodological section.

 >Regarding the functional enrichment, the choice of the pathway database is not clear. Why is Reactome chosen for genes annotation and KEGG for metabolites?

  >Taking into account that breast cancer related biological findings is not the scope of this manuscript, a deeper analysis of biological information revealed by network blocks is necessary: which pathways are block-specific? Which pathway or pathways are present in the bigger block?  Do the pathway distances between blocks have any relation with previous knowledge about breast cancer?

 > Furthermore, the pathway distances within and between blocks are computed just for the lower level of blocks. Since the network is hierarchical, pathway distances can be obtained for each level. This would indicate if the hierarchy is biological meaningful too. Moreover, the same genes are known to be annotated to related pathways. Therefore, this expected result does not represent a validation to determine the biological meaning of the blocks in the network. A good approach could be to select a gene X and see if the rest of the genes of the pathways, where gene X is annotated, are in the same block of gene X (or in near blocks in the hierarchy). And repeat this for a high number of genes.

 >Finally, the edge confidence score looks like important to improve the network characterization, however they do not use this score to optimize the final network. I was expecting the comparison between the original SBM-based network and the corrected SBM-based network via edge confidence score optimization. In order to use the edge confidence scores, a way to compute thresholds for missing and spurious edges should be proposed.

 > Resuming, SBM-based biological networks are a new way to represent omics data and it represents a novel approach for SBMs. However, a novel application should be coupled with a suitable interpretation of its results. Moreover, the main conclusion or this manuscript is not clear and the main question for a new methodological development is unanswered, why should I use this method?

*Response:*

*We thank the reviewers for the nice summary of our work. In order to sharpen the focus, we clarified in the abstract and at the end of the introduction that our goal is to pave the ground for the usage of the SBM model for different types of biomolecular networks. Investigating the capability of SBMs for representing and analysing different types of biological networks was the key challenge addressed in our article. We did not intend to compare the networks between layers, but rather assess to which extent the SBM is applicable to derive useful information in terms of (i) relevant clustering as well as (ii) network-based, alternative edge scores.*

*We have shown that a lot more SBM-predicted blocks have biological counterparts (i.e. more genes or metabolites associated with certain Reactome or KEGG terms are clustered together) than expected by chance, and we show in our revised version that biological processes known to be relevant to the examined phenotype can be derived (new Figs. 4, 5, Table 2). In addition, we showed that the SBM-based edge relevance scores coincide with the correlation values (which have not been given to fit the network to the SBM). These results support our hypothesis that the SBM is suitable to represent and analyze biomolecular networks in which interactions are derived from correlations. This opens the avenue to new types of analyses using the SBM and its output for which this work lays the foundation.*

*We hope that the reviewers will also find that the revised version supports this reasoning and the derived conclusion.*

*In response to other points that were raised only here but not in the specific points include below:*

*To improve the clarity of the description of the data usage from the beginning, we reformulated the according sentences in the abstract and introduction which now read:*

*‘We apply SBM-based analysis independently to three correlation-based networks of breast cancer data originating from high-throughput measurements of different molecular layers: transcriptomics, proteomics, or metabolomics.’*

*‘Here we showcase the SBM-based analysis (overview in Fig. 1A) for three networks of different molecular types, derived from either transcriptomic, proteomic or metabolomics data of breast cancer tumours.’*

*In order to facilitate the understanding of the different SBM versions, we added an explanation on the hierarchical and degree-corrected version of the SBM into the methods part and comment on the relationship between underfitting and the hierarchical SBM in the revised methods section.*

**>Specific points:**

**>1) Data preprocessing:**

 Why are the three datasets used? Is it just a matter of different complexity? Datasets are not completely characterized as the number of features is not indicated.

*Response:*

*Protein, mRNA and metabolites are key molecules in cells, which are widely applied, in isolation or in combinations, in different biomedical research domains. Their abundance and interconnectedness in networks are therefore of high interest if aiming to characterize cells or tumorous tissue. We used them to illustrate three examples for cellular interaction networks, whose interactions have different biological meanings. Moreover, in the revised article now we explain that we use these networks to reflect different characteristics of tumorous tissue/cells.*

*The number of features (i.e. number of mRNAs, proteins and metabolites) are given in the network characteristics in Table 1 (‘entities’ before NA removal and afterwards). In order to improve accessibility to the reader, we also state these numbers in the methods part of the revised manuscript and clearly identify them as feature count (‘mRNA, protein, metabolite data for ER-breast cancer tumors’).*

 >Authors say missing values of metabolite data were imputed previously. It is not clear whether they performed the imputation or not. In case they did, the algorithm used is not indicated. If missing values of metabolite data were imputed, why weren't the missing values of protein data?

*Response:*

*We used the metabolite data as provided in Budczies et al. 2009 in which imputation had been performed. In order to clarify this, we reformulated the sentence in the methods part to ‘The metabolite data did not contain missing values as imputation had been performed in the original publication ^[Budczies et al. 2009]^.’*

*We used the processed protein data as provided in the original publication (Mertins et al, 2014). In general, in contrast to missing values in metabolite data which are usually considered to occur due to abundance below detection limit, missing values in protein data can have multiple different reasons making imputation less straightforward. Because we do not focus on data pre-processing here, we kept the data as close as possible to those originally published and rather took the opportunity to provide an idea of how to incorporate datasets with missing data into our proposed analysis framework.*

**>2) Network generation and reduction:**

 They use different test-multiple correction rates for mRNA, protein and metabolites in order to obtain a similar degree of reduction. The authors should demonstrate this does not lead to any biases. The reduction objective is set at 10 ^7^edges, is there any reason?

*Response:*

*We reformulated and describe this part in more detail in the revised manuscript. In fact, we intended to provide examples for different corrections methods and thresholds which are frequently used, and to achieve a high degree of reduction (to reduce runtime for SBM fit) while still ensuring that the resulting biomolecular network is as connected as possible (see Fig. 1C). Similarity of the degree of reduction between the three data layers was not a goal to achieve. The border of 10 ^7^ edges arose out of computation time considerations (runtime scales with edge count for SBM fit in graph-tool, they correspond to several hours and Gbs of memory consumption for a single initialization), and thus could be adapted to each user’s case.*

 >In Figure 1.D, a linear relationship is expected for the blue dots but it not perfectly linear, why? Could the scale-free reduction be modified to improve it?

*Response:*

*Usually, a perfect fit to scale-freeness cannot be achieved because altering the cut-off threshold leads to removal of multiple edges at the same time; the achievable combinations of which edges belong to the network are fully determined by the correlation values associated to the edges. We show possible scale-free fit indices (that index is a measure of how closely the node degree distribution resembles a power-law, i.e. how close the network is to scale-freeness) of the networks reduced by edge thresholding in new Fig. 1D. Therein, it also becomes clear that other thresholds could be used and can lead to ‘better’ scale-freeness in the resulting networks – while at the same time making the resulting network less connected. Other specific requirements on the degree of scale-freeness can be adapted on a case-by-case basis.*

**>3) Stochastic block models:**

 How are the number of initial partitions (500) defined? Is there any relation between the suitable number of initial partitions and the network complexity (size)?

*Response:*

*The number of initializations was chosen as first assessment whether this is sufficient to be able to distinguish between the different SBM types – which was the case. As is clear, for some networks different SBM types are more closely related whereas for others, they are well more separated. This will depend on the actual network/data that is fitted and serves as orientation. Of course, more initializations are always better, but it is subject to a trade-off between computation time and finding a good partition.*

**>4) Functional enrichment:**

 They use Reactome database for genes annotation and KEGG for metabolites, but there is no an explanation of why that choice.

*Response:*

*We added the explanation to the revised version. Reactome provides a hierarchical annotation which qualifies it for comparison to the hierarchical structure given by the SBM, KEGG is one of the annotation databases used most for metabolites.*

 >Figure 3.C is confusing. I do not understand the objective of this analysis. In order to demonstrate the biological meaning of the hierarchical SBM, the pathway distances comparison within and between blocks at every hierarchical level (as in Figure 3.B) would be better than the provided analysis.

*Response:*

*We altered the accompanying description of Fig. 3C. What we intended is to relate the distance of two SBM blocks in the SBM hierarchy (x-axis) to the distances between the Reactome terms associated to the blocks (y-axis). For a matching hierarchy structure of Reactome and SBM, we would expect a positive correlation, i.e. more distant SBM blocks in the SBM hierarchy having more distantly related Reactome terms. We do not find convincing evidence for this.*

*To clarify the approach, we restructured Fig. 3 (by moving the results for the second distance measure into the supplement, new Fig. S3).*

*In addition, we now also include the analysis of within-block distance vs. between-block distances for the other hierarchy levels as suggested by the reviewer (new Fig. 3D). As in our previous analysis in Fig. 3C, we cannot find strong evidence for the hierarchy of the SBM coinciding with the Reactome hierarchy. Please note that the numbers of blocks with annotation get low in higher hierarchy levels, thereby reducing the number of hierarchy levels that can be considered for this analysis.*

 >As mentioned before, a deeper biological interpretation of the network is necessary.

 I am not sure what the clustering analysis is contributing to. Modules obtained by WGCNA shows good results looking at the number significant of pathways, but are they biologically relevant? Are they related to breast cancer?

*Response:*

*We provide additional biological interpretations of the clustering results in the revised version of the manuscript (see new Figs. 4, 5, Table 2). They show that the SBM-derived clustering can detect biological pathways known to be implicated in breast cancer according to oncogenic signatures from MSigDB, such as extracellular matrix organization or the cell cycle (see new Fig. 4), or fatty acid biosynthesis (Table 2). In addition, the relevance of further processes can be predicted, e.g. chromatin organization (new Fig. 5).*

# Peer review: Reviewer response for version 1

**DOI:** 10.5256/f1000research.20484.r47223

**Published:** 2019-04-29

**Authors:** Silvia Pineda San Juan

**Affiliations:** 1 Division of Transplant Surgery, Department of Surgery, University of California, San Francisco (UCSF), San Francisco, CA, USA; 2 Centro Nacional de Investigaciones Oncológicas (CNIO), Madrid, Spain

The manuscript provides an innovative way of using stochastic block models to perform networks for different omics data.

 The idea is interesting but the application should be better characterized. I was expecting a network integrating the different datasets, but the network is performed separately per each dataset. This fact should be addressed at the very beginning to avoid confusion to the readers. 

 Also, the use of the three datasets is not justified, why these three and no others? 

 It looks like the authors want to compare the three networks applied to the three datasets (mRNA, proteins, and metabolites) but the different sample size among other factors makes them not comparable.

 Finally, one of my main concern is that there is not a clear conclusion of the study, are they proposing a network that is better than the ones that already exist? (this is not assessed). Are they finding new biological insights for breast cancer? (this is not shown). The final conclusion is not clear since the authors did not give a biological example of the method application. It is not clear if the method outperforms others or if the method is able to find new biological interpretations, etc. Why people should use this method? What type of information will they obtain?

More specific points:

 Data preparation and network generation:

- It is not clear to me why the authors used mRNA, protein and metabolites for this network study. Is it just because they were available, or is there a hypothesis under this selection? Why don't they use other omics data available in the TCGA? The sample size is pretty reduced for the proteomics data with only 36 samples. The other thing I do not understand is why they used metabolomics data measured in other individuals. And for the mRNA they do not give an exact sample size. 
- Why do they use Bonferroni for mRNA expression and Benjamini-Hochberg for protein, metabolite and just 0.01 for protein? This should be better justified.
- They replace 0 values by NA, why? There is a big difference between a lack of expression and a missing value.
- Do they filter for those genes that have a very low expression among samples? They only specify this for protein data, but what about mRNA expression? Are they considered only 0 for low expression or a very small cut-off normally used in mRNA analysis?  
- Regarding the scale-free reduction. They explain that the technique removes weak links until met a criterion based on WGCNA package, but it does not well explain the process for this and how they applied this to the data. Please explain. 

Fitting SBM:

- They built the network based on a stochastic block model (SBM) representation, the nodes of a network are partitioned into blocks according to their similarity in connectivity. It is not clear how the SBM is applied to the data and how the similarities are obtained. 

For the SBM representing biological function:

- The whole module is unclear to me since they do not provide any biological or functional interpretation. I don't understand the goal of this. Figure 3 is also quite confusing.

I confirm that I have read this submission and believe that I have an appropriate level of expertise to confirm that it is of an acceptable scientific standard, however I have significant reservations, as outlined above.

# Response

**Published:** 2019-08-12

**Authors:** Katharina Baum

**Affiliations:** Max Delbrück Center for Molecular Medicine in the Helmholtz Association, Germany

We thank Dr. Silvia Pineda San Juan for the careful revision of our manuscript. Her comments helped us enhancing the clarity and stringency of our presentation and the reader's accessibility to our work. We respond to her specific comments and describe the introduced changes to version 2 of our manuscript below.

 >The manuscript provides an innovative way of using stochastic block models to perform networks for different omics data.

 The idea is interesting but the application should be better characterized. I was expecting a network integrating the different datasets, but the network is performed separately per each dataset. This fact should be addressed at the very beginning to avoid confusion to the readers. 

*Response:*

*We thank the reviewer for her appreciation of our idea. In order to improve the clarity of the description of the data usage from the beginning, we reformulated the corresponding sentences in the abstract and introduction which now read:*

*‘We apply SBM-based analysis independently to three correlation-based networks of breast cancer data originating from high-throughput measurements of different molecular layers: either transcriptomics, proteomics, or metabolomics.’*

*‘Here we showcase the SBM-based analysis (overview in Fig. 1A) for three networks of different molecular types, derived from either transcriptomic, proteomic or metabolomics data of breast cancer tumours.’*

 >Also, the use of the three datasets is not justified, why these three and no others? 

 It looks like the authors want to compare the three networks applied to the three datasets (mRNA, proteins, and metabolites) but the different sample size among other factors makes them not comparable.

 Finally, one of my main concern is that there is not a clear conclusion of the study, are they proposing a network that is better than the ones that already exist? (this is not assessed). Are they finding new biological insights for breast cancer? (this is not shown). The final conclusion is not clear since the authors did not give a biological example of the method application. It is not clear if the method outperforms others or if the method is able to find new biological interpretations, etc. Why people should use this method? What type of information will they obtain?

*Response:*

*In order to sharpen the focus, we clarified in the abstract and at the end of the introduction that our goal is to pave the ground for the usage of the SBM model for different types of biomolecular networks. Investigating the capability of SBMs for representing and analysing different types of biological networks was the key challenge addressed in our article. We did not intend to compare the networks between layers, but rather assess to which extent the SBM is applicable to derive useful information in terms of (i) relevant clustering as well as (ii) network-based, alternative edge scores.*

*We have shown that a lot more SBM-predicted blocks have biological counterparts (i.e. more genes or metabolites associated with certain Reactome or KEGG terms are clustered together) than expected by chance, and biological processes known to be relevant to the examined phenotype are can be derived (new Fig. 4, 5, Table 2). In addition, we showed that the SBM-based edge relevance scores coincide with the correlation values (which have not been given to fit the network to the SBM). These results support our hypothesis that the SBM is suitable to represent and analyze biomolecular networks in which interactions are derived from correlations.*

*This opens the avenue to new types of analyses using the SBM for which this work lays the foundation.*

*To strengthen our findings, as suggested by the reviewer, we now also include more biological interpretations of the clustering results (see Figs. 4, 5, Table 2). They show that the SBM-derived clustering can detect biological pathways known to be implicated in breast cancer according to oncogenic signatures from MSigDB, such as extracellular matrix organization and the cell cycle (see new Fig. 4), or fatty acid biosynthesis (Table 2). In addition, the relevance of further processes can be predicted, e.g. the chromatin organization (new Fig. 5).*

>More specific points:

 >Data preparation and network generation:  

 It is not clear to me why the authors used mRNA, protein and metabolites for this network study. Is it just because they were available, or is there a hypothesis under this selection? Why don't they use other omics data available in the TCGA? The sample size is pretty reduced for the proteomics data with only 36 samples. The other thing I do not understand is why they used metabolomics data measured in other individuals. And for the mRNA they do not give an exact sample size. 

*Response:*

*Protein, mRNA and metabolites are key molecules in cells, which are widely applied, in isolation or in combinations, in different biomedical research domains. Their abundance and interconnectedness in networks are therefore of high interest if aiming to characterize cells or tumorous tissue. We used them to illustrate three examples for cellular interaction networks, whose interactions have different biological meanings. Moreover, in the revised article now we explain that we use these networks to reflect different characteristics of tumorous tissue/cells.*

*Other potential data types could be e.g. mutations, copy number variation, DNA methylation or miRNAs, which are interesting avenues to further explore. While they could be useful, there are some caveats associated with them, e.g.: the derived interactions within layers are even less directly interpretable than for mRNA, protein or metabolite; networks generated from mutations and copy number variation are extremely sparse; the functional interpretation of DNA methylation data relies on transcription and the resulting networks are extremely big; the roles of miRNAs are less well known. Therefore, we decided to restrict our analyses to the three biomolecular entities mRNA, proteins and metabolites. We included these considerations when introducing the employed data layers in the results part of the revised manuscript.*

*Concerning the metabolite data: We are convinced that metabolites characterize a highly interesting layer of intra-tumor processes which is complementary to the gene expression associated layers mRNA and proteins. Unfortunately, metabolomics have not been measured for TCGA samples which is why we resorted to an alternative cohort for this data layer.*

*Please note that the number of tumour samples for each data layer were stated in the methods section (mRNA: 237, protein: 36, metabolite: 68). We now included that value for the mRNA data layer also into the main text.*

 >Why do they use Bonferroni for mRNA expression and Benjamini-Hochberg for protein, metabolite and just 0.01 for protein? This should be better justified.

*Response:*

*We explain our approach for network reduction more in detail in the methods and results part of the revised version of the manuscript. In fact, at first, we applied both Bonferroni and Benjamini-Hochberg correction methods, both of which are widely used and accepted, along with the classical significance thresholds 0.01 and 0.05 to the networks of all three data layers (see Fig. 1C). We finally chose the correction method and threshold for each data layer considering a trade-off between minimal network size (i.e. minimal computation time for the subsequent fit to SBM) and maximal connectedness of the reduced network: We used the combination which provided a high degree of reduction (less than 10 million edges in the network) while maximizing the size of the largest connected component in the network. While the stringent Bonferroni correction is necessary to achieve a sufficient degree of reduction for the mRNA network, it severely disrupted the connectedness for the protein and metabolite data layer leading to less than 30% or 65% of the nodes being in the largest connected component for protein or metabolite, respectively (see Fig 1C).*

*Using different correction methods and significance thresholds serve as examples of typical scenarios which could be envisioned during network reduction. Finally, every user could use their own thresholds reasonable for network reduction.*

 >They replace 0 values by NA, why? There is a big difference between a lack of expression and a missing value.

*Response:*

*We replaced NAs by -10 in the log-counts of the RNAseq data. As stated in the methods part, the reason for this is the following: The RNAseq data are logarithmized (relative) counts. In this case the NAs are therefore artefacts from logarithmizing a zero count. Our replacement served to reverse this artefact.*

 >Do they filter for those genes that have a very low expression among samples? They only specify this for protein data, but what about mRNA expression? Are they considered only 0 for low expression or a very small cut-off normally used in mRNA analysis?  

*Response:*

*Apart from the replacement of NAs by very small values (-10 in log-counts) in the mRNA data, we use the data as provided by TCGA. The rationale for this is to reduce bias with respect to which nodes we consider. Since we do not focus on expression strength, but on the connection between molecular species, i.e. co-variation of expression, also lowly abundant species could, and indeed do, play a role, i.e. they have non-zero degree in the reduced networks. We dedicate new Figure S1D to an illustration of this fact.*

*One can imagine multiple other criteria of entity removal prior to analysis (e.g. tissue-specific GTEx, using pathways of interest) but this is not the focus of our work.*

 >Regarding the scale-free reduction. They explain that the technique removes weak links until met a criterion based on WGCNA package, but it does not well explain the process for this and how they applied this to the data. Please explain. 

*Response:*

*We incorporated a more detailed explanation of this reduction technique in the methods part and added an additional panel to Fig. 1 (Fig. 1D) which illustrates intermediate results of the process. This is the revised methods part: ‘For the reduction by imposing a scale-free architecture of the reduced network, we employed the *pickHardThreshold *function of the WGCNA package (Langfelder et al, 2008) with the default requirement (0.85) on goodness of fit to a power-law degree distribution of the nodes. Given the symmetric absolute correlation matrix of the network edges, this function reduces the network by one of a given set of edge thresholds at a time and determines the scale-free fit index R ^2^ which lies between 0 (bad fit) and 1 (perfect fit) by comparing the resulting degree distribution of the reduced network to a power-law degree distribution. The lowest of the tested edge thresholds that gives a scale-free fit index > 0.85 is reported as estimated threshold. For the edge thresholds, we started with a grid with stepsize 0.05 between 0.3 and 0.95, refining according to the resulting estimates to vectors with stepsize 0.001 between 0.5 and 0.625 for the mRNA network, between 0.7 and 0.82 for the protein network and between 0.3 and 0.4 for the metabolite network. Finally estimated edge correlation thresholds were 0.603 (mRNA), 0.788 (protein), and 0.375 (metabolite).’*

 >Fitting SBM:  

 They built the network based on a stochastic block model (SBM) representation, the nodes of a network are partitioned into blocks according to their similarity in connectivity. It is not clear how the SBM is applied to the data and how the similarities are obtained. 

*Response:*

*We extended the corresponding section describing the SBM in the Methods, in which we now also summarize the equations underlying the building of the SBM and the relationship between model likelihood and properties of the network graph derived from the data.*

 >For the SBM representing biological function:  

 The whole module is unclear to me since they do not provide any biological or functional interpretation. I don't understand the goal of this. Figure 3 is also quite confusing.

*Response:*

*The assumption behind the analysis is that a predicted block structure is meaningful if many of the predicted blocks have a biological counterpart, e.g., biological pathway. Such an analysis was performed as a starting point for assessing potential biological relevance. Nevertheless, because we agree that additional biological interpretations would benefit the reader, we included other examples of biological interpretations (new Figs. 4, 5, Table 2 and accompanying description in the results section). In addition, in order to improve accessibility, we reduced the contents of Fig. 3 (by only showing results for one distance measure) and clearly indicated which SBM hierarchy level the examined blocks stem from.*

# Peer review: Reviewer response for version 1

**DOI:** 10.5256/f1000research.20484.r47226

**Published:** 2019-04-26

**Authors:** Lei Xie, Yue Qiu

**Affiliations:** 1 Department of Computer Science, Hunter College, The City University of New York, New York, NY, USA; 2 City University of New York, New York, NY, USA

The paper by Baum et al describes a method to construct biological networks from omics data using stochastic block models (SBMs). The application of SBM on mRNA, protein and metabolic data gives a new way of deriving information from correlation based biological networks. The proposed method could be a useful addition to network biology. To strengthen the manuscript, I would suggest the following points to be addressed:

1. In the over representation analysis, while the statistics on distance of Reactome/KEGG terms shows the block generated here is significantly better than random. Is it possible to provide a such comparison between the clustering result by SBM and WGCNA?
2. In the network reduction step, a linear relationship is expected between log-frequency and log-node-degree. While the scale-free fit index (R ^2^) will be > 0.85 with WGCNA default requirement, how different threshold affect that linearity is not clear from Fig 1D. Also, how well can the network be reduced by significance of correlation fit to a scale free network?
3. To demonstrate that SBM can provide biological insight, more detailed analysis on the benchmark data sets could be useful. For example, what are unique and common pathways for different breast cancer subtypes, how well are these findings consistent with existing knowledge?
4. Figure 4 shows SBM based confidence score can be used to predict the existence of an edge. However, it is not clearly stated how the edges are determined as putatively missing or spurious. Also, the description for figure 4A is hard to understand. 

We confirm that we have read this submission and believe that we have an appropriate level of expertise to confirm that it is of an acceptable scientific standard, however we have significant reservations, as outlined above.

# Response

**Published:** 2019-08-12

**Authors:** Katharina Baum

**Affiliations:** Max Delbrück Center for Molecular Medicine in the Helmholtz Association, Germany

We thank Prof. Lei Xie and Yue Qiu for their comments that helped improving the accessibility of the presented contents and strengthened the manuscript. In the following, we answer to the specific points they raised and how we addressed them in version 2.

 >The paper by Baum et al describes a method to construct biological networks from omics data using stochastic block models (SBMs). The application of SBM on mRNA, protein and metabolic data gives a new way of deriving information from correlation based biological networks. The proposed method could be a useful addition to network biology. To strengthen the manuscript, I would suggest the following points to be addressed:  

 >In the over representation analysis, while the statistics on distance of Reactome/KEGG terms shows the block generated here is significantly better than random. Is it possible to provide a such comparison between the clustering result by SBM and WGCNA?

*Response:*

*We thank the reviewers for their appreciation of our work.We now also provide the computation of the SBM distances for the mRNA and protein WGCNA clustering results (see new Fig. 3F and new Fig. S3C). They show also a bigger distance between clusters than within clusters. We added the resulting p-values to the comparison of the distances for both SBM (Fig. 3B, D) and WGCNA (Fig. 3F, S3C) approach.*

 >In the network reduction step, a linear relationship is expected between log-frequency and log-node-degree. While the scale-free fit index (R2) will be > 0.85 with WGCNA default requirement, how different threshold affect that linearity is not clear from Fig 1D. Also, how well can the network be reduced by significance of correlation fit to a scale free network?

*Response:*

*Fig. 1D (now 1E) has been included for illustrative purposes only to show some further characteristics (in this case: degree distribution) apart from those in Table 1 of the six networks employed for the SBM-based analysis. It can be used to appreciate the fact that the networks reduced by requirement on significance of correlation are not optimized for it but still may exhibit a close to linear relationship between log-frequency and log-node-degree. For clarification, we added the scale-free fit indices obtained for each network into old Fig. 1D - now Fig. 1E.*

*We also extended the description of the scale-freeness fitting procedure and approach by using the WGCNA package (see revised Methods section). Please note that less strict correlation thresholds than the employed here lead to networks being further away from a network with scale-free characteristic (this is inherent to the WCGNA fitting approach as the least stringent threshold is determined leading to a scale-free fit index of at least 0.85) and more stringent correlation thresholds can lead to networks being closer to a scale-free network (i.e. to higher values of R ^2^). We give now these dependencies for the three networks in new Fig. 1D.*

*Please note that reducing by a threshold on significance of correlation is a different reduction method and it is neither expected nor intended that the resulting networks are scale-free. In order to illustrate the relationship between the two reduction methods, we introduced new illustrations in supplemental figure S1. Therein,*

*- we show in new panel S1A that for the metabolite and mRNA layer, due to the datasets not having NA values and thus the sample size being the same for each and every pair of metabolite or mRNA species, the p-values of the correlation being different from zero depend monotonously on the absolute correlation values. This is not the case for the protein dataset for which different sample sizes may occur. Therefore, the correlation value for some interactions will be backed by fewer data points only which leads to increased p-value despite the same correlation value.*

*- we show in new S1B that, for the mRNA and metabolite data layer, the scale-free fit index for networks reduced by different significance thresholds can be directly compared to those from Fig. 1D.*

*- we show in new S1C how the significance threshold of correlation being different from zero relates to the scale-free score for the protein dataset and where the four networks reduced by the four considered significance thresholds locate therein.*

 >To demonstrate that SBM can provide biological insight, more detailed analysis on the benchmark data sets could be useful. For example, what are unique and common pathways for different breast cancer subtypes, how well are these findings consistent with existing knowledge?

*Response:*

*We agree that a comparison of results between breast cancer subgroups to provide biological insights into the utility of the clustering would be useful. However, our work focusses on determining whether SBMs are suitable to represent biomolecular networks as they have not been explored for molecular network analysis.*

*We indeed expect additional and complementary biological findings to what is already known with the SBM approach. To strengthen our work, we also added some showcase examples of biological interpretation (new Figs. 4, 5, Table 2 and Description in the text). They show that the SBM-derived clustering can detect biological pathways known to be implicated in breast cancer according to oncogenic signatures from MSigDB, such as extracellular matrix organization or the cell cycle (see new Fig. 4), or fatty acid biosynthesis (Table 2). In addition, the relevance of further processes can be predicted, e.g. the chromatin organization (new Fig. 5).*

 >Figure 4 shows SBM based confidence score can be used to predict the existence of an edge. However, it is not clearly stated how the edges are determined as putatively missing or spurious. Also, the description for figure 4A is hard to understand. 

*Response:*

*We extended the description of the edge detection by adding the following sentences in the section ‘Assessing edge relevance by SBM-based edge confidence scores’:*

*‘For the six reduced networks, all edges that exist in the network were considered as ‘putatively spurious’. Similarly, all edges that were not in the network because they had been removed from the (fully connected) correlation-based network during the reduction procedure were considered as ‘putatively missing’.’*

*We clarified the legend to new Fig. 6A (old Fig. 4A) that now reads:*

*‘(A) Histograms of edge confidence scores for the best fitting SBM of the three networks reduced by criterion on scale-freeness. Top: spurious edge confidence scores, computed for all edges existing in the network; middle and bottom: missing edge confidence scores, computed for all edges that were removed during the reduction procedure (middle: missing edges between nodes with degree>0 in the reduced network, bottom: missing edges adjacent to a node of degree zero in the reduced network).’*
